# From Plant to Yeast—Advances in Biosynthesis of Artemisinin

**DOI:** 10.3390/molecules27206888

**Published:** 2022-10-14

**Authors:** Le Zhao, Yunhao Zhu, Haoyu Jia, Yongguang Han, Xiaoke Zheng, Min Wang, Weisheng Feng

**Affiliations:** 1 School of Pharmacy, Henan University of Chinese Medicine, Zhengzhou 450046, China; 2 Co-Construction Collaborative Innovation Center for Chinese Medicine and Respiratory Diseases by Henan and Education Ministry of P. R. China, Henan University of Chinese Medicine, Zhengzhou 450046, China; 3 College of Chemistry and Materials Engineering, Beijing Technology and Business University, Beijing 100048, China; 4 Beijing Key Laboratory of Plant Research and Development, Beijing Technology and Business University, Beijing 100048, China

**Keywords:** *Artemisia annua*, artemisinin, synthetic biology, genetic engineering, transcription factor

## Abstract

Malaria is a life-threatening disease. Artemisinin-based combination therapy (ACT) is the preferred choice for malaria treatment recommended by the World Health Organization. At present, the main source of artemisinin is extracted from *Artemisia annua*; however, the artemisinin content in *A. annua* is only 0.1–1%, which cannot meet global demand. Meanwhile, the chemical synthesis of artemisinin has disadvantages such as complicated steps, high cost and low yield. Therefore, the application of the synthetic biology approach to produce artemisinin in vivo has magnificent prospects. In this review, the biosynthesis pathway of artemisinin was summarized. Then we discussed the advances in the heterologous biosynthesis of artemisinin using microorganisms (*Escherichia coli* and *Saccharomyces cerevisiae*) as chassis cells. With yeast as the cell factory, the production of artemisinin was transferred from plant to yeast. Through the optimization of the fermentation process, the yield of artemisinic acid reached 25 g/L, thereby producing the semi-synthesis of artemisinin. Moreover, we reviewed the genetic engineering in *A. annua* to improve the artemisinin content, which included overexpressing artemisinin biosynthesis pathway genes, blocking key genes in competitive pathways, and regulating the expression of transcription factors related to artemisinin biosynthesis. Finally, the research progress of artemisinin production in other plants (*Nicotiana*, *Physcomitrella*, etc.) was discussed. The current advances in artemisinin biosynthesis may help lay the foundation for the remarkable up-regulation of artemisinin production in *A. annua* through gene editing or molecular design breeding in the future.

## 1. Introduction

Plants fix carbon through photosynthesis and use solar energy to convert water and CO_2_ into organic matter to produce primary metabolites, which are used as precursors to synthesize a series of secondary metabolites through secondary metabolism. These secondary products are not only important for plant adaptation and response to the environment, but are also the source of many clinical drugs [1]. According to the chemical structure and biosynthesis pathway, plant secondary metabolites can be divided into three major groups: terpenoids (54%), alkaloids (27%), phenolics (18%) and others (1%) [2]. Terpenoids are the largest number of plant secondary products, possessing diversified structures and functions. Based on the number of 5-carbon isoprene units, terpenoids are classified into hemiterpenoids (5C), monoterpenoids (10C), sesquiterpenoids (15C), diterpenoids (20C), sesterterpenoids (25C), triterpenoids (30C), tetraterpenoids (40C), and polyterpenoids (>40C) [3]. Paclitaxel (taxol), a diterpenoid anticancer drug isolated from *Taxus brevifolia*, has been approved for the clinical treatment of breast cancer, non-small cell lung cancer, ovarian cancer and AIDS-related Kaposi sarcoma (used as second-line therapy) [4]. Many plant polyphenolics metabolites, such as flavonoids, phenolic acids, lignans and tannins, are powerful antioxidants. Curcumin, a polyphenol compound that is produced by *Curcuma longa*, has antioxidant, anti-inflammatory and anticancer properties [5]. Among other types of plant secondary metabolites, an example is melatonin, which is derived from tryptophan and was initially isolated from animals before being discovered in plants. Melatonin plays an important role in plant growth and development as well as the response to biotic and abiotic stress; in addition, it has strong antioxidant activity [6].

Artemisinin is a plant secondary metabolite of sesquiterpenoids, which was first isolated from the medicinal plant *Artemisia* *annua* L. by Tu Youyou in 1972 [7]. Artemisinin-based combination therapy (ACT) is currently the preferred treatment for malaria recommended by the World Health Organization (WHO). Malaria is a life-threatening disease caused by the *Plasmodium parasite*, which is transmitted to humans through the bite of infected female *Anopheles* mosquitoes. So far, the global malaria epidemic remains severe. According to the latest *World malaria report 2021*, malaria infection cases in 2020 were found in 85 countries, infecting more than 241 million people and causing 627,000 deaths, mainly in Africa, Southeast Asia and the Eastern Mediterranean. Compared with 2019, there were 14 million more malaria infections and 47,000 more deaths than in 2020 due to the disruption of medical services during the COVID-19 pandemic [8]. In 2015, Tu Youyou was awarded the Nobel Prize in Physiology and Medicine for her discovery of a new method to treat malaria with artemisinin. Recent studies have found that artemisinin and its derivatives have anti-cancer, antibacterial and immunomodulatory properties—in addition to the efficacy of treating malaria [9]. Moreover, artemisinin can also be used for the treatment of diabetes [10] and pulmonary tuberculosis [11], which have broad application prospects.

The content of artemisinin in *A. annua* is very low, being only 0.1–1% DW (dry weight) of the plant. The process of extracting artemisinin from *A. annua* is complicated and costly, which cannot meet the global demand for artemisinin [12]. Although the chemical synthesis of artemisinin was successful as early as 1983, it has not been put into commercial production due to the disadvantages of having multiple steps, a high cost and low yield [13]. Therefore, how to obtain artemisinin efficiently, economically and in large quantities has become an international research hotspot. With the development of molecular biology, the application of synthetic biology to produce active components of medicinal plants has broad application prospects [14]. In this review, the artemisinin biosynthesis pathway was summarized, then reconstituting the biosynthesis pathway of artemisinin using microorganisms as chassis cells was discussed, and finally, the research progress in the production of artemisinin from different plants was reviewed, which laid the foundation for the mass, rapid and low-cost acquisition of artemisinin.

## 2. The Artemisinin Biosynthesis Pathway

**Figure 1:**
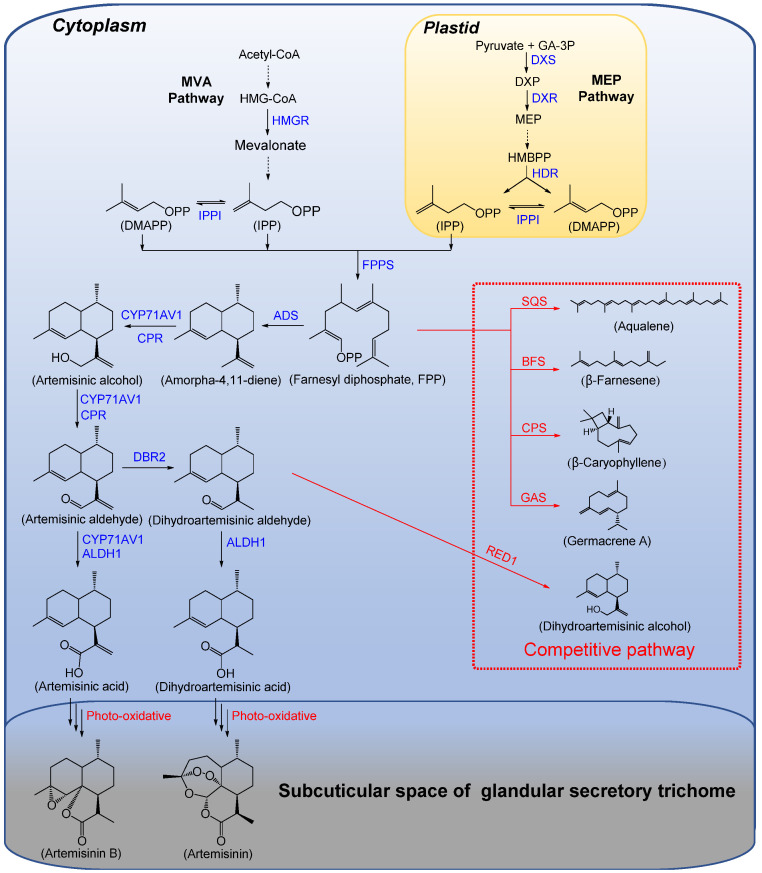
Artemisinin biosynthesis pathway and competitive metabolic pathway in *A. annua*. The biosynthesis of artemisinin is derived from the MVA and MEP pathways. FPP is the important intermediate at the branch point of artemisinin biosynthesis, which can not only be converted into amorpha-4,11-diene and artemisinin, but also into other terpenoids through competitive metabolic pathways, such as squalene, β-farnesene, β-caryophyllene, germacrene A, etc. The competitive pathways are marked with red boxes. HMG-CoA, 3-hydroxy-3-methylglutaryl coenzyme A; GA-3P, glyceraldehyde 3-phosphate; DXP, 1-deoxy-D-xylulose 5-phosphate; MEP, 2-C-methyl-D-erythritol-4-phosphate; HMBPP, 4-hydroxy-3-methyl-but-2-enyl diphosphate; IPP, isopentenyl diphosphate; DMAPP, dimethylallyl diphosphate; HMGR, 3-hydroxy-3-methylglutaryl coenzyme A reductase; DXS, 1-deoxy-D-xylulose 5-phosphate synthase; DXR, 1-deoxy-D-xylulose 5-phosphate reductoisomerase; HDR, 4-hydroxy-3-methyl-but-2-enyl diphosphate reductase; IPPI, isopentenyl diphosphate isomerase; FPPS, farnesyl diphosphate synthase; ADS, amorpha-4,11-diene synthase; CYP71AV1, cytochrome P450 monooxygenase; CPR, cytochrome P450 reductase; ALDH1, aldehyde dehydrogenase 1; DBR2, artemisinic aldehyde Δ11(13) reductase; SQS, squalene synthase; BFS, β-farnesene synthase; CPS, β-caryophyllene synthase; GAS, germacrene A synthase; RED1, dihydroartemisinic aldehyde reductase.

In order to generate artemisinin in microorganisms and plants with the synthetic biology method, the biosynthesis pathway of artemisinin needs to be dissected. Artemisinin was derived from the plant terpenoid biosynthesis pathway by using IPP (isopentenyl diphosphate) and its isomer, DMAPP (dimethylallyl diphosphate), as raw materials, which were produced by the MVA (mevalonate) pathway in the cytoplasm and the MEP (methylerythritol phosphate) pathway in the plastid. Derivatives of the MVA pathway were precursors to sesquiterpenes, triterpenes and polyterpenoids, while the MEP pathway was generally involved in the biosynthesis of monoterpenes, diterpenes and tetraterpenes [15]. Two molecules of IPP and one molecule of DMAPP were catalyzed by FPPS (farnesyl diphosphate synthase) to form 15-carbon FPP (farnesyl diphosphate), which entered the downstream metabolic pathway of artemisinin biosynthesis (Figure 1).

ADS (Amorpha-4,11-diene synthase) catalyzed the cyclization of FPP to form amorphine-4,11-diene, the precursor of artemisinin [16]. Under the coordination synergistic effect of CYP71AV1 (cytochrome P450 monooxygenase) and CPR (cytochrome P450 reductase), amorpha-4,11-diene was further oxidized to artemisinic alcohol and artemisinic aldehyde [17,18]. Artemisinic aldehyde was catalyzed by CYP71AV1 and ALDH1 (aldehyde dehydrogenase 1) to generate artemisinic acid [19]. Starting from artemisinic aldehyde, an additional branch emerged. Artemisinic aldehyde was catalyzed by DBR2 (artemisinic aldehyde Δ11(13) reductase) to generate DHAAA (dihydroartemisinic aldehyde), and DHAAA was converted to DHAA (dihydroartemisinic acid) by ALDH1 [20]. Artemisinic acid and DHAA were thought to be the final compounds formed by the enzyme-catalyzed reactions in this pathway (Figure 1). In the subcuticular space of glandular-secreting trichomes (GSTs) on the surface of *A. annua* leaves, artemisinic acid was converted to artemisinin B and DHAA to artemisinin through photo-oxidation, which are a series of non-enzymatic reactions [21,22].

At present, artemisinin has been produced by the synthetic biology approach, mostly through the semi-synthetic route. The precursors of artemisinin, such as amorpha-4,11-diene, artemisinic acid and DHAA, were prepared in microorganisms by metabolic engineering, and then artemisinin was generated by chemical synthesis; however, some studies suggested that there may be a specifically expressed and highly active peroxidase in the GSTs of *A. annua*, which can catalyze the conversion of DHAA to artemisinin [23]. If such a peroxidase existed, the total biosynthesis of artemisinin would be carried out in microorganisms, rather than semi-synthesis.

## 3. Metabolic Engineering in Microorganisms

With the advancement of sequencing technology, more and more medicinal plant genomes have been sequenced, and the biosynthetic pathways of many natural products have been elucidated [24]. On this basis, the biosynthetic pathway of natural products can be reconstructed in microorganisms, and natural products and their precursors are able to be obtained rapidly and in large quantities through biological fermentation, thereby laying a foundation for the commercial application of natural plant products. As an effective antimalarial drug, the development of artemisinin was restricted by the cost of extracting compounds from natural *A. annua* and the complexity of chemical synthesis; however, for those who are most affected by malaria (such as African areas), classical plant breeding may not be enough to reduce the production cost of artemisinin to an affordable price. Therefore, using microorganisms (*Escherichia coli* and *Saccharomyces cerevisiae*) as chassis cells, the heterologous biosynthesis of artemisinin or its precursor through metabolic engineering may provide an alternative source of artemisinin supply. It took Keasling’s laboratory ten years to achieve the semi-synthesis of artemisinin, thereby transferring the production of artemisinin from plants to microorganisms.

In 2003, Martin et al. transformed the codon-optimized *ADS* gene into *E. coli*, simultaneously co-expressed the *E. coli* SOE4 operon (encoding the rate-limiting enzyme genes *dxs*, *ippHp* and *ispA* of the *E. coli* MEP pathway), and heterologously expressed the MVA pathway genes (pMevT and pMBIS plasmids) from *S. cerevisiae*. This was the first time that amorpha-4,11-diene, the precursor of artemisinin, was synthesized in *E. coli* at a concentration of 24 mg/L [25] (Table 1). After optimization of the fermentation conditions, the yield of amorpha-4,11-diene increased 20-fold to 0.5 g/L [26]. Tsuruta et al. continued to optimize the genes of the yeast MVA pathway for heterologous expression in *E. coli* and replaced the yeast *HMGS* and *tHMGR* genes on the pMevT plasmid with more active equivalent genes from *Staphylococcus aureus*. Combined with the restricted supply of carbon and nitrogen, the amorpha-4,11-diene production in *E. coli* reached 27.4 g/L [27]. On the basis of the heterologous expression of the yeast MVA pathway genes (pAM92 plasmid), the *ADS*, *AMO* (amorpha-4,11-diene oxidase) and *CPR* genes of *A. annua* were simultaneously expressed to produce artemisinic acid in *E. coli* with the highest yield being 105 mg/L [28].

In the downstream biosynthetic pathway of artemisinin, the conversion from amorphine-4,11-diene to artemisinic acid and DHAA required the synergistic catalysis of CYP71AV1 and CPR, both of which were associated with the cell’s endomembrane system; however, *E. coli* lacked the CYP450 gene and the endomembrane system of eukaryotic cells, resulting in the affected expression of *CYP71AV1* and *CPR* genes. In 2006, Ro et al. modified the FPP biosynthesis pathway in *S. cerevisiae* to prompt FPP production, which down-regulated the expression of *EGR9* and inhibited the conversion of FPP to squalene, thus allowing more FPP to flow into the artemisinic acid biosynthesis pathway. Meanwhile, the *ADS*, *CYP71AV1* and *CPR* genes of *A. annua* were transformed into yeast strain EPY224 (Table 1). Finally, the biosynthesis of artemisinic acid was achieved in yeast with a yield of 100 mg/L [18]. Moreover, the biosynthesized artemisinic acid was able to be secreted outside of the engineered yeast, facilitating the purification of artemisinic acid from the yeast fermentation broth, which further prompted the industrialization process of artemisinin.

Westfall et al. adopted the yeast strain, CEN.PK2, to overexpress all the genes of the MVA pathway while down-regulating the *EGR9* gene so that the production of amorpha-4,11-diene reached 40 g/L [30]. In 2013, Paddon et al. transformed the *ADS*, *CYP71AV1*, *CPR1*, *CYB5*, *ADH1* and *ALDH1* genes of *A. annua* into yeast after codon optimization to reduce the accumulation of artemisinic aldehyde and improved the fermentation process. As a result, the yield of artemisinic acid reached 25 g/L (Table 1), which met the demand of industrial production. Then, artemisinic acid was extracted from the fermentation broth, and after four steps of chemical synthesis in vitro, artemisinin was finally acquired with an overall yield of 40–45% [31].

In summary, the use of synthetic biology methods to produce artemisinic acid in microorganisms mainly made the following improvements: 1. Codon optimization. The key genes of artemisinic acid biosynthesis pathway were codon-optimized and transformed into *E. coli* and yeast. 2. Reconstruction of metabolic pathways and selection of enzymes with high catalytic activity. Enzymes of the yeast MVA pathway were replaced with enzymes from *S. aureus*. 3. Choice of suitable chassis cells. The CYP450 oxidase and reductase of *A. annua* were expressed in eukaryotic yeast. 4. Control of the flow of metabolites. In yeast cells, the down-regulation of the *ERG9* gene (encoding squalene synthase) inhibited the conversion of FPP to squalene. Meanwhile, the genes of the MVA pathway were overexpressed under strong promoters, thereby allowing more FPP to flow into the artemisinic acid biosynthesis pathway.

## 4. Production of Artemisinin in Genetically Engineered Plants

### 4.1. Genetic Engineering in A. annua

The artemisinin content in *A. annua* can be improved by overexpressing artemisinin biosynthetic genes, blocking key genes in competitive pathways of artemisinin biosynthesis, or regulating the expression of transcription factors involved in artemisinin biosynthesis.

#### 4.1.1. Overexpression of Key Genes in Artemisinin Biosynthesis

In the past two decades, key genes in the artemisinin biosynthesis pathway have been isolated and characterized (Figure 1). The content of artemisinin in *A. annua* was adjusted by regulating the expression of genes involved in the MVA and MEP pathways, or by overexpressing key genes in the downstream biosynthesis pathway of artemisinin (Table 2).

According to the genetic map and genomic data of *A. annua*, *HMGR* and *DXR* genes are closely related to artemisinin content [32,33]. HMGR and DXR are the rate-limiting enzymes of the MVA and MEP pathway, which provided the substrates IPP and DMAPP for the biosynthesis of artemisinin. By applying competitive inhibitors (fosmidomycin and mevinolin) to suppress the activities of DXR and HMGR, respectively, it was found that the production of artemisinin decreased by 14.2% and 80.4%, indicating that the MVA pathway was the main carbon donor for artemisinin biosynthesis [34]. The *HMGR* gene from *Catharanthus roseus* was overexpressed in *A. annua* through *Agrobacterium*-mediated transformation, and the artemisinin content of transgenic plants reached 0.60 mg/g DW, while artemisinin in non-transgenic lines was only 0.37 mg/g DW [35]. Overexpression of the *DXR* gene from *C. roseus* in *A. annua* prompted a 2.33-fold increase in the yield of artemisinin to 1.21 mg/g DW [36].

Overexpression of the *IPPI1* and *HDR1* genes in *A. annua* were able to increase the accumulation of artemisinin and artemisinin B, while inhibition of the expression of the *HDR1* gene reduced the artemisinin content [37,38]. FPP was the precursor of secondary metabolites such as sesquiterpenes (including artemisinin), triterpenes, coenzyme Q, plastoquinone, etc., so increasing the production of FPP can promote the biosynthesis of artemisinin. The *FPPS* gene of *A. annua* was first isolated by Matsushita in 1996 [39], and overexpression of the *FPPS* gene increased the yield of artemisinin by 1.38–3.36 times [40,41,42].

ADS catalyzed the conversion of FPP to amorpha-4,11-diene, the first key enzyme in the artemisinin biosynthesis pathway, and the *ADS* gene was specifically expressed in the GST cells of *A. annua* [43]. The haplotype genome comparison of *A. annua* revealed that the copy number of the *ADS* gene was highly correlated with artemisinin yield [44]. Overexpression of the *ADS* gene significantly increased not only artemisinin content, but also the content of artemisinic acid and DHAA [45]. DBR2 was another key enzyme in the artemisinin biosynthesis pathway, which was also specifically expressed in the GSTs of *A. annua*. DBR2 catalyzed artemisinic aldehyde to form dihydroartemisinic aldehyde, which further generated artemisinin. If the activity of DBR2 decreased, more artemisinic aldehyde was converted into artemisinic acid, resulting in an increased accumulation of artemisinin B [20]. The content of artemisinin in DBR2-overexpressed transgenic plants was 1.50–2.14 mg/g DW, which was 1.59–2.26 times that of the control [46].

The overexpression of a single key gene involved in the artemisinin biosynthesis can increase the artemisinin content, but the increase was not significant. Therefore, simultaneous overexpression of two or more key genes was able to remarkably improve the content of artemisinin [33]. When *HMGR* and *ADS* genes were overexpressed in *A. annua*, the artemisinin content was increased by 8.65-fold to 1.73 mg/g DW [47]. The *ADS* and *FPPS* genes were fused together and overexpressed under the regulation of the CaMV35S or CYP71AV1 promoter. The CaMV35S promoter significantly up-regulated the expression of the *ADS* and *FPPS* genes, but the accumulation of artemisinin did not increase significantly; however, under the control of the CYP71AV1 promoter, the *ADS-FPPS* fusion gene was specifically expressed in GSTs and significantly enhanced the artemisinin concentration to 26 mg/g DW [48]. Co-expression of the *CYP71AV1* and *CPR* genes can improve the content of artemisinin by 1.38–2.68 times, reaching 0.98–2.44 mg/g DW [36,49]; when the three genes, *FPPS*, *CYP71AV1* and *CPR,* were co-expressed, the artemisinin content was increased by 3.6 times, reaching 2.98 mg/g FW [50]; when the four genes, *ADS*, *CYP71AV1*, *CPR* and *ALDH1,* were co-expressed, the artemisinin content reached 27 mg/g DW, which was 3.4 times that of the control [51].

Therefore, the use of transgenic methods to regulate the expression of key genes in the biosynthesis pathway of artemisinin, and the overexpression of one or more key genes, can remarkably improve the content of artemisinin in transgenic plants (Table 2), which was of great significance for obtaining new varieties of *A. annua* with a high artemisinin content.

**Table 2:** Genetic engineering to increase artemisinin production in *A. annua*.

Gene Name	Expression Mode	Artemisinin (mg/g DW)		Fold Change	Reference
		Control	Transgenic		
**MVA or MEP pathway key genes**					
*CrHMGR* ^1^	Overexpression	0.32	0.39	1.22	[52]
		0.37	0.60	1.62	[35]
*CrDXR* ^1^	Overexpression	0.52	1.21	2.33	[36]
*IPPI1*	Overexpression	0.75	2.5	3.33	[38]
*HDR1*	Overexpression	0.05	0.09	1.8	[37]
*HDR1*	Down-regulation	0.05	0.03	0.6	
*GaFPPS* ^2^	Overexpression	3	10.08	3.36	[42]
*FPPS*	Overexpression	6.5	0.9	1.38	[40]
		5.3	13.3	2.5	[41]
**Artemisinin biosynthesis pathway key genes**					
*ADS*	Overexpression	0.6	1.2	2	[45]
*DBR2*	Overexpression	0.94	2.14	2.26	[46]
		8	22.6	2.83	[53]
*ALDH1*	Overexpression	8	25.6	3.2	[54]
*HMGR*, *FPPS*	Overexpression	5	9	1.8	[55]
*HMGR*, *ADS*	Overexpression	0.2	1.73	8.65	[47]
*ADS-FPPS* fusion	Overexpression	10	26	2.6	[48]
*CYP71AV1*, *CPR*	Overexpression	0.91	2.44	2.68	[36]
		0.71 (mg/g FW)	0.98 (mg/g FW)	1.38	[49]
*FPPS*, *CYP71AV1*, *CPR*	Overexpression	0.83 (mg/g FW)	2.98 (mg/g FW)	3.6	[50]
*ADS*, *CYP71AV1*, *CPR*	Overexpression	6.4	15.1	2.4	[56]
*ADS*, *CYP71AV1*, *CPR*, *ALDH1*	Overexpression	8	27	3.4	[51]
**Competitive pathway key genes**					
*SQS*	Down-regulation	10	31.4	3.14	[57]
*CPS*	Down-regulation	2.32	3.6	1.55	[58]
*CPS*	Down-regulation	6.26	11.08	1.77	[59]
*BFS*	Down-regulation	6.26	11.08	1.77	
*GAS*	Down-regulation	6.26	12.71	2.03	
*SQS*	Down-regulation	6.26	10.70	1.71	

^1^*CrHMGR* and *CrDXR* genes were from *C. roseus*. ^2^ *GaFPPS* gene was from *Gossypium arboreum*.

#### 4.1.2. Suppression of Competitive Metabolic Pathways

FPP has different metabolic flows in plants. It can not only be converted into amorpha-4,11-diene and enter the artemisinin biosynthesis pathway, but can also be converted into terpenoids such as β-farnesene, β-caryophyllene, germacrene A, squalene and *epi*-cedrol, which enter the biosynthesis pathway of other secondary metabolites (Figure 1). Therefore, by suppressing the flow of FPP into other competing pathways, more FPP can enter the artemisinin biosynthesis pathway and increase the yield of artemisinin [60,61] (Table 2).

SQS (squalene synthase) was the first key enzyme that catalyzed the sterol biosynthesis pathway. Using the hairpin RNA-mediated RNAi method to inhibit the expression of the *SQS* gene, the content of sterol was reduced by 37–58%, and the content of artemisinin was significantly increased, reaching 31.4 mg/g DW—3.14 times that of the control [57].

CPS (β-caryophyllene synthase) catalyzed the conversion of FPP to β-caryophyllene [62]. When the antisense RNA approach was adopted to down-regulate the expression of the *CPS* gene, the expression levels of the genes related to the artemisinin biosynthesis pathway were significantly up-regulated in transgenic plants. The results showed that the content of β-caryophyllene decreased by 40–62.7%, while the content of artemisinin increased by 54.9% [58].

Through RNA interference, the key enzyme genes, *CPS*, *BFS*, *GAS* and *SQS,* involved in the competitive pathway of artemisinin biosynthesis were gene-silenced. The results showed that the expression levels of key genes related to the artemisinin biosynthesis pathway (*ADS*, *CYP71AV1*, *DBR2*, *ALDH1*) were up-regulated, and the contents of artemisinin and DHAA were significantly increased. Compared with non-transgenic plants, the content of artemisinin in anti-CPS, anti-BFS, anti-GAS and anti-SQS transgenic plants rose by 77%, 77%, 103% and 71%, respectively [59].

In the biosynthesis pathway of artemisinin, RED1 (dihydroartemisinic aldehyde reductase) competed with ALDH1 to bind dihydroartemisinic aldehyde so that dihydroartemisinic aldehyde was converted into dihydroartemisinic alcohol, which means that artemisinin cannot be produced (Figure 1). If the expression level of the *RED1* gene was reduced, more dihydroartemisinic aldehyde can enter the artemisinin biosynthesis pathway [63]. Ranjbar et al. analyzed the content of artemisinin in eight *Artemisia* species and the changes in the expression levels of key genes in the biosynthesis pathway of artemisinin. The results indicated that the content of artemisinin in *A. annua* was the highest, followed by *Artemisia absinthium*. This may be because the expression levels of the *ADS* and *DBR2* genes in *A. annua* were higher than those in the other seven *Artemisia* species, while in *A. absinthium*, the expression of the *ALDH1* gene was down-regulated and the expression of the *RED1* gene was up-regulated, which reduced the artemisinin content in *A. absinthium* [64].

#### 4.1.3. Regulation of Transcription Factors Expression

In the secondary metabolism of plants, transcription factors (TFs) can regulate the expression of a series of genes in metabolic pathways, and overexpression or suppression of these TFs is able to effectively regulate the accumulation of plant secondary metabolites [60,61]. A variety of TFs have been identified to be related to the biosynthesis of artemisinin, including WRKY, bHLH, AP2/ERF, bZIP, MYB, etc. (Table 3).

##### WRKY TF Family

WRKY TFs are one of the largest TF families in plants, with a conserved WRKYGQK sequence at the N-terminus and a typical zinc finger protein structure at the C-terminus [65]. WRKY TFs participate in the regulation of plant growth, development, senescence and response to environmental stress [66]. It has been reported that AaWRKY1, AaWRKY4, AaWRKY9, AaWRKY17, AaGSW1 and AaGSW2 are involved in the regulation of artemisinin biosynthesis (Table 3).

AaWRKY1 was the first cloned TF of *A. annua* [67]. AaWRKY1 activates the expression of the *ADS*, *CYP71AV1* and *DBR2* genes, and AaWRKY1 takes part in the jasmonic acid (JA) signaling pathway [68]. Overexpression of the AaWRKY1 gene increased the content of artemisinin by 1.3–2.0 times [60]. In AaWRKY4-overexpressed plants, the expression levels of the *ADS*, *CYP71AV1*, *DBR2* and *ALDH1* genes were significantly improved, and the production of artemisinin was increased by 35–50% [69]. AaWRKY9 was specifically expressed in the GSTs of *A. annua*, which responded to both light and JA signals, and positively regulated the biosynthesis of artemisinin. AaWRKY9 can bind to the promoters of the *AaDBR2* and *AaGSW1* genes to up-regulate the expression of artemisinin biosynthesis pathway genes, and the artemisinin production was increased by 1.6–2.2 times in AaWRKY9-overexpressed plants [70]. AaWRKY17 was also a TF that positively regulated artemisinin biosynthesis. Moreover, AaWRKY17 can activate the expression of two defense marker genes, *PR5* (Pathogenesis-Related 5) and *NHL10* (NDR1/HIN1-LIKE 10), and improve the tolerance of *A. annua* to *Pseudomonas syringae*, so AaWRKY17 could be used in the transgenic breeding of *A. annua* to improve artemisinin content and resistance to pathogens [71].

AaGSW1 (GLANDULAR TRICHOME-SPECIFIC WRKY 1) is a GSTs specific WRKY TF, which is regulated by AaMYC2 in the JA signaling pathway and AabZIP1 in the ABA signaling pathway. Overexpression of AaGSW1 activated the transcript levels of the *ADS*, *CYP71AV1*, *DBR2*, *ALDH1* and *ORA* genes, and improved the content of artemisinin by 55–100% [72]. A recent study reported that AaGSW1 can bind to the promoters of TF AaTCP15 (teosinte branched1/cycloidea/proliferating 15) and AaORA (octadecanoid-derivative responsive AP2-domain protein), forming the AaGSW1-AaTCP15/AaORA module network that regulates artemisinin biosynthesis through JA and ABA signaling transduction [73]. When AaGSW2 was overexpressed in *A. annua*, the density of GSTs was significantly increased in the overexpressed plants, and the artemisinin content went up 2-fold compared with the control [74].

##### bHLH TF Family

bHLH TFs are a class of TFs containing the basic helix-loop-helix (bHLH) domain that recognizes the E-box motif (CANNTG) of the promoter region [75]. According to the genome data, the bHLH TFs in *A. annua* have nearly 200 members, which makes it one of the largest TF families in *A. annua* [33]. So far, it has been reported that AabHLH1, AaMYC2 and AabHLH112 positively regulate the biosynthesis of artemisinin, while AabHLH2 and AabHLH3 are the negative regulators in artemisinin accumulation (Table 3).

AabHLH1 can bind to the promoters of the *ADS* and *CYP71AV1* genes and positively regulate artemisinin biosynthesis. In AabHLH1-overexpressed plants, the expression levels of the *ADS*, *CYP71AV1*, *DBR2* and *HMGR* genes are significantly up-regulated [76], and the artemisinin content is increased 1.3-fold [77]. Moreover, the expression of AabHLH1 is induced by JA and AabHLH1 interacted with all nine AaJAZ proteins, so AabHLH1 is also involved in the regulation of JA-induced artemisinin biosynthesis [77].

AaMYC2 belongs to the bHLH TF superfamily and is the core factor of the JA signaling pathway, which can bind to the G-box motif of the *CYP71AV1* and *DBR2* gene promoters. Overexpression of AaMYC2 remarkably improved the transcription levels of the *CYP71AV1* and *DBR2* genes, and the content of artemisinin increased by 23.55% compared with the control, while in the RNAi plants of AaMYC2, the artemisinin content decreased to 54.81% of the control [78].

As a low-temperature-inducible TF, AabHLH112 positively regulates artemisinin biosynthesis through AaERF1. Yeast one-hybrid results showed that AabHLH112 cannot bind to the promoters of artemisinin biosynthesis-related genes, but could bind to the promoter of the *AaERF1* gene [79]. AaERF1 is a member of the AP2/ERF TF family and can up-regulate the expression of artemisinin biosynthesis genes [80]. Overexpression of AabHLH112 significantly activated the transcript of the *AaERF1* gene, which further promoted the expression of artemisinin biosynthesis genes. In AabHLH112-overexpressed plants, the artemisinin content reached 14.35 mg/g DW, which increased by 70.42% compared to the control (8.42 mg/g DW) [79].

AabHLH2 and AabHLH3 are MYC-type bHLH TFs, which suppress the expression of the *ADS* and *CYP71AV1* genes by antagonizing AaMYC2 and participate in the negative regulation of artemisinin biosynthesis. Compared with the control, the artemisinin accumulation decreased by 27–66% and 21–61% in the overexpressed plants of AabHLH2 and AabHLH3, respectively. In contrast, the artemisinin content was increased by 42–87% and 35–60% in AabHLH2 and AabHLH3 RNAi plants, respectively [81]. As negative regulators, AabHLH2 and AabHLH3 may be good targets for prompting artemisinin production in *A. annua* by gene editing.

**Table 3:** Transcription factors involved in artemisinin biosynthesis pathway.

Name	Function	Expression Mode	Fold Change of Artemisinin Content	Reference
**WRKY TF family**				
AaWRKY1	AaWRKY1 activates the expression of *ADS*, *CYP71AV1*, *DBR2* genes.	Overexpression	1.30–2.00	[68]
AaWRKY4	AaWRKY4 prompts the expression of *ADS*, *CYP71AV1*, *DBR2*, *ALDH1* genes.	Overexpression	1.35–1.50	[69]
AaWRKY9	AaWRKY9 positively regulates the expression of *AaDBR2* and *AaGSW1* genes.	Overexpression	1.60–2.20	[70]
		Down-regulation	0.55–0.65	
AaWRKY17	AaWRKY17 activates the transcription of *ADS*, *PR5* and *NHL10* genes and enhances the resistance of *A. annua* to the pathogen *P. syringae*.	Overexpression	1.50–1.87	[71]
		Down-regulation	0.79–0.85	
AaGSW1	AaGSW1 is a GST-specific TF, and positively regulates the expression of *CYP71AV1* and *AaORA* genes.	Overexpression	1.55–2.00	[72]
AaGSW2	As a GST-specific TF, AaGSW2 positively regulates the initiation of GST.	Overexpression	2.00	[74]
		Down-regulation	0.54	
**bHLH TF family**				
AabHLH1	AabHLH1 activates transcription of *ADS* and *CYP71AV1* genes and responds to JA induction.	Overexpression	1.30	[77]
		Down-regulation	0.60	
AaMYC2	AaMYC2 prompts the expression of *CYP71AV1* and *DBR2* genes and responses to JA induction.	Overexpression	1.24	[78]
		Down-regulation	0.55	
AabHLH112	AabHLH112 promotes artemisinin biosynthesis by activating TF AaERF1.	Overexpression	1.70	[79]
AabHLH2	AabHLH2 and AabHLH3 suppress the expression of *ADS* and *CYP71AV1* genes by antagonizing AaMYC2, and negatively regulate artemisinin biosynthesis.	Overexpression	0.34–0.73	[81]
		Down-regulation	1.42–1.87	
AabHLH3		Overexpression	0.39–0.79	
		Down-regulation	1.35–1.60	
**AP2/ERF TF family**				
AaERF1	AaERF1 and AaERF2 activate the transcription of *ADS* and *CYP71AV1* genes and respond to JA signaling.	Overexpression	1.19–1.67	[80]
AaERF2		Overexpression	1.24–1.51	
AaORA	AaORA is a trichome-specific TF, which promotes the expression of *ADS*, *CYP71AV1*, *DBR2* and *AaERF1* genes, and improves the resistance of *A. annua* to the pathogen *B. cinerea*.	Overexpression	1.40–1.53	[82]
		Down-regulation	0.64–0.52	
AaTAR1	AaTAR1 activates the transcription of *ADS* and *CYP71AV1* genes and regulates trichome development.	Overexpression	1.22–1.38	[83]
		Down-regulation	0.36–0.61	
**bZIP TF family**				
AabZIP1	AabZIP1 activates the transcription of *ADS* and *CYP71AV1* genes through ABA signaling, indirectly promotes the expression of *AaDBR2* and *AaALDH1* genes through AaMYC2 and improves the drought resistance of *A. annua*.	Overexpression	1.40–1.60	[84,85]
AabZIP9	AabZIP9 activates the expression of *ADS* gene and positively regulates artemisinin biosynthesis.	Overexpression	1.23–1.67	[86]
AaHY5	AaHY5 positively regulates light-induced artemisinin biosynthesis through interacting with TF AaGSW1.	Overexpression	2.00	[87]
		Down-regulation	0.50	
AaTGA6	AaTGA6 takes part in artemisinin biosynthesis regulated by SA signaling and promotes artemisinin biosynthesis by activating the TF AaERF1.	Overexpression	1.90–2.20	[88]
		Down-regulation	0.40–0.80	
**MYB TF family**				
AaMYB1	AaMYB1 participates in the biosynthesis of artemisinin and GA and regulates the trichome development.	Overexpression	2.00	[89]
AaMIXTA1	AaMIXTA1 is a GST-specific TF, which regulates GST formation and cuticle biosynthesis.	Overexpression	2.00	[90]
		Down-regulation	0.75	
AaTAR2	AaTAR2 positively regulates GST development, as well as artemisinin and flavonoid biosynthesis.	Overexpression	~1.38–1.54 ^1^	[91]
		Down-regulation	~0.57–0.84 ^1^	
AaMYB16 (AaMIXTA-like 2)	AaMYB5 and AaMYB16 show the antagonism and regulate the development of GST by competitively binding to the *AaHD1* promoter to form AaHD1-AaMYB5 or AaHD1-AaMYB16 complexes.	Overexpression	1.34–1.56	[92]
		Down-regulation	0.73–0.81	
AaMYB5		Overexpression	0.73–0.85	
		Down-regulation	1.45–1.84	
AaMYB17	AaMYB17 is a GST-specific TF and positively regulates artemisinin biosynthesis.	Overexpression	1.88	[93]
		Down-regulation	0.75	
AaMYB15	As a GST-specific TF, AaMYB15 negatively regulates the biosynthesis of artemisinin by inhibiting the expression of *AaORA* gene.	Overexpression	~0.60–0.74 ^1^	[94]
		Down-regulation	~1.52 ^1^	
AaTLR1	AaTLR1 and AaTLR2 interact with AaWOX1 to form the AaTLR1-AaWOX1-AaTLR2 complex to negatively regulate GST formation and artemisinin biosynthesis.	Overexpression	0.51–0.88	[95]
		Down-regulation	1.32–1.84	
AaTLR2		Overexpression	0.57–0.81	
**O** **t** **her TF family**				
AaZFP1	As a C2H2-type TF, AsZFP1 activates the transcription of *AaIPPI1* gene and prompts artemisinin biosynthesis.	Overexpression	1.60	[96]
AaSPL9	AaSPL9 positively regulates glandular trichomes initiation and artemisinin biosynthesis by activating TF AaHD1.	Overexpression	1.33–1.60	[97]
AaSEP4	AaSEP4, as a MADS TF, promotes artemisinin biosynthesis by directly activating TF AsGSW1.	Overexpression	1.19–1.72	[98]

^1^ The change fold was calculated according to the data in the references.

##### AP2/ERF TF Family

The APELATA 2/ethylene response factor (AP2/ERF) TFs are one of the most important TF families in plants, and are involved in plant responses to biotic and abiotic stresses, as well as regulating various plant developmental and secondary metabolism processes [99]. AP2/ERF TF family members all contain the AP2 domain consisting of about 60 amino acid residues. According to the number of AP2 domains and whether they contain other domains, the AP2/ERF TF family is divided into AP2, ERF, DREB, RAV and Soloist 5 subfamilies [100,101].

AaERF1 and AaERF2 (Table 3), which respond to JA signaling, activate the transcription of the *ADS* and *CYP71AV1* genes by binding to the CBF2 and RAA motifs of these two gene promoters. Compared with the wild type, overexpression of AaERF1 prompted an increase in yield of artemisinin by 19–67% and overexpression of AaERF2 by 24–51% [80].

AaORA (Table 3) is a trichome specific AP2/ERF TF, which is expressed in GSTs and non-glandular T-shaped trichomes (TSTs). In AaORA-overexpressed plants, the expression levels of the *ADS*, *CYP71AV1*, *DBR2* and *AaERF1* genes are all significantly up-regulated, and the contents of artemisinin and DHAA are increased by 40–53% and 22–35%, respectively [82].

In addition, overexpression of both AaERF1 and AaORA activated the transcription of defense marker genes, *PDF1.2* (PLANT DEFENSIN1.2) and *B-CHI* (BASIC CHITINASE), thereby enhancing the resistance of *A. annua* to the pathogenic fungus *Botrytis cinerea* [82,102]. A recent study reported that AaORA formed a transcriptional complex with the TF AaTCP14, which prompted the expression of *DBR2* and *ALDH1* genes, leading to an increased accumulation of artemisinin, and the AaORA-AaTCP14 complex was induced and activated by JA [103].

AaTAR1 (TRICHOME AND ARTEMISININ REGULATOR 1) (Table 3), as an AP2/ERF TF, directly binds to CBF2 and RAA motifs, activates the expression of the *ADS* and *CYP71AV1* genes, and participates in the positive regulation of glandular trichome development and artemisinin and cuticular wax biosynthesis. The contents of artemisinin and DHAA were enhanced by 22–38% and 69–130% in AaTAR1-overexpressed plants, respectively [83].

##### bZIP TF Family

bZIP (basic leucine zipper) TFs contain two specific motifs, one that is located at the C-terminal, which is composed of basic amino acids for binding to the target DNA sequence, and the other that is located at the N-terminal with the leucine zipper motif, which is required for the dimerization of bZIP TFs [104]. bZIP TFs participate in the regulation of plant growth and development to cope with various environmental stresses [105].

There are 75 bZIP family members in the *A. thaliana* genome, which are divided into 10 groups based on sequence similarity and conserved motifs [106]. The group A AtbZIP proteins contain ABRE (abscisic acid-responsive element) *cis*-elements, which are involved in ABA signal transduction [107]. ABA-dependent bZIP TFs can bind conserved DNA sequences with ACGT core *cis*-elements, such as ABRE (ACGTGG/TC), G-box (CACGTG), etc. [108]. AabZIP1, AabZIP9, AaHY5 and AaTGA6 are reported to be positive regulators of artemisinin biosynthesis (Table 3).

ABA treatment can prompt the artemisinin accumulation in *A. annua* [109]. AabZIP1 is a member of the group A bZIP family, and its expression is induced by abiotic stresses such as ABA, drought and high salt. AabZIP1 can bind to the ABRE *cis*-element of the *ADS* and *CYP71AV1* gene promoters through the N-terminal C1 domain and activate the expression of the *ADS* and *CYP71AV1* genes. When AabZIP1 is overexpressed, the artemisinin content in transgenic plants showed a 1.5-fold increase [84].

A recent study reported that AabZIP1 can also directly bind to the promoter of the *AaMYC2* gene and up-regulate the expression of AaMYC2, while AaMYC2 bound to the promoters of the *AaDBR2* and *AaALDH1* genes, which indicated that AabZIP1 indirectly activated the expression of the *AaDBR2* and *AaALDH1* genes through AaMYC2 and further improved artemisinin biosynthesis [85]. In addition, AabZIP1 can directly activate the transcription of cuticle wax biosynthesis genes *AaCER1* and *AaCYP86A1* and enhance the drought resistance of *A. annua* [85]. The results showed that the AabZIP1–AaMYC2 transcriptional module was a cross-talk between the ABA and JA signaling pathways in artemisinin biosynthesis, which provided a useful candidate gene for the genetic breeding of *A. annua* with a high artemisinin content and strong drought resistance.

As a member of the group C bZIP family in *A. annua*, AabZIP9 can bind to the “ACGT” *cis*-element of the *ADS* and *CYP71AV1* gene promoters to up-regulate the expression of the *ADS* gene. Overexpression of AabZIP9 prompted the content of artemisinin and DHAA to increase by 23.2–67.1% and 34.5–92.8%, indicating that AabZIP9 was a positive regulator of artemisinin biosynthesis [86].

AaHY5, a member of the group H of bZIP TFs, regulated light-induced artemisinin biosynthesis by interacting with AaGSW1 in the WRKY family. When AaHY5 was overexpressed, the contents of artemisinin and DHAA were up-regulated approximately 2-fold [87].

SA (salicylic acid) treatment prompted the accumulation of artemisinin [110], and AaTGA6, a member of group D bZIP TFs, was involved in the artemisinin biosynthesis regulated by the SA signal [88]. As an important regulator of the SA signaling pathway, AaNPR1 up-regulated the expression of *AaTGA6*. AaTGA6 directly bound to the “TGACG” element of the *AaERF1* gene promoter and activated the expression of key genes in artemisinin biosynthesis (*ADS*, *CYP71AV1*, *DBR2*, *ALDH1*). The artemisinin content was increased by 90–120% in AaTGA6-overexpressed plants, while AaTGA3 inhibited the binding of AaTGA6 to the *AaERF1* gene promoter [88].

##### MYB TF Family

The MYB TFs contain a highly conserved DNA-binding domain: the MYB domain, which consists of about 52 amino acid sequence repeats (Rs), each of which form three α-helices [111]. According to the number of adjacent repeats, the MYB TFs can be divided into four classes: R2R3-MYB, 1R-MYB, 3R-MYB and 4R-MYB [111]. R2R3-MYB TFs mainly participate in cell morphogenesis [112], phytohormone signaling response [113], secondary metabolism regulation [114] and response to environmental stress [115]. The reported MYB TFs of *A. annua* all belong to the R2R3-MYB class. AaMYB1, AaMIXTA1, AaTAR2, AaMYB16 (AaMIXTA-like 2) and AaMYB17 positively regulate artemisinin biosynthesis, while AaMYB5, AaMYB15, AaTLR1 and AaTLR2 are the negative regulators in artemisinin accumulation (Table 3).

AaMYB1 promoted the glandular trichome initiation and artemisinin accumulation. Overexpression of AaMYB1 improved the density of glandular trichome, up-regulated the key genes of artemisinin biosynthesis, and significantly increased the content of artemisinin. Furthermore, both AaMYB1 and its orthologue, AtMYB61, participated in the regulation of trichome and root development, stomatal aperture and gibberellin biosynthesis [89]. AaMIXTA1 is predominantly expressed in the basal cells of GSTs and takes part in GSTs formation and cuticle biosynthesis. Overexpression of AaMIXTA1 remarkably improved the accumulation of artemisinin [90]. AaTAR2 is also involved in the initiation and development of GSTs and regulated the secondary metabolism of terpenoids and flavonoids in *A. annua*. Overexpression of AaTAR2 significantly up-regulated the expression of key genes in artemisinin and flavonoid biosynthesis pathways, such as *HMGR*, *DXS*, *CYP71A1*, *DBR2* and *PAL*, *C4H*, *CHS*, *F3H*, and *DFR*. Furthermore, the HD-ZIP (homeodomain-leucine zipper) TFs, AaHD1 and AaHD8, prompted the transcription of *AaTAR2* by binding to the L1 box (TAAAGATA) of the *AaTAR2* promoter [91]. AaMYB17 was specifically expressed in GSTs of shoot tips. In AaMYB17-overexpressed *A. annua* plants, the density of GSTs was enhanced by 1.3–1.6 times, and the content of artemisinin was increased from 8 mg/g to 15 mg/g. In AaMYB17 RNAi plants, the GSTs density and artemisinin content were significantly reduced [93].

AaMYB15 is also a GSTs-specific TF, but unlike the previous R2R3-MYB TFs, AaMYB15 participates in the negative regulation of artemisinin accumulation. Overexpression of AaMYB15 resulted in the significant suppression of the expression of key genes for artemisinin biosynthesis, such as *ADS*, *CYP71AV1*, *DBR2* and *ALDH1*, and ultimately reduced the artemisinin content. The opposite results were observed in antisense-AaMYB15 *A. annua* plants. Yeast one-hybrid results indicated that AaMYB15 could not bind to the promoters of these key genes, but rather directly bound to the promoters of *AaORA*. AaORA is an AP2/ERF TF that positively regulates artemisinin biosynthesis, and AaMYB15 inhibited artemisinin biosynthesis by suppressing the expression of the *AaORA* gene [94].

AaMYB5 and AaMYB16 are two antagonistic MYB TFs of *A. annua*, which regulate the development of GSTs. Overexpression of AaMYB5 inhibited the initiation of GSTs, resulting in the decline in artemisinin content, while AaMYB16 had the opposite effect; however, none of them could independently regulate the formation of GSTs, but played a regulatory role by competitively binding to the *AaHD1* gene promoter to form AaHD1–AaMYB5 or AaHD1–AaMYB16 complexes [92]. AaHD1 was an HD-ZIP TF that promoted the initiation of GSTs by directly activating the transcription of *AaGSW2*. In addition, JA was also associated with the AaHD1–AaMYB5/AaMYB16 regulatory network, which regulates the transcription of glandular trichome development-related genes through the AaJAZ8 protein [92].

A recent study reported two MYB TFs that negatively regulated GSTs development and artemisinin production: AaTLR1 and AaTLR2. Overexpression of AaTLR1 and AaTLR2 decreased the artemisinin content by 11.5–49.4% and 19–43%, respectively, while knockdown of the *AaTLR1* and *AaTLR2* genes resulted in the opposite effect. Yeast three-hybrid results showed that AaTLR1 and AaTLR2 interacted with AaWOX1 (WUSCHEL homeobox 1) to form the AaTLR1–AaWOX1–AaTLR2 complex, which negatively regulated the initiation of GSTs [95].

##### Other TF Families

In addition to the TFs discussed above, there are other TFs that also participate in the biosynthesis of artemisinin, such as AaZFP1, AaSPL9 and AaSEP4, all of which are positive regulators.

AaZFP1 (zinc finger protein 1), as a C2H2-type TF, positively regulates the transcription of the *AaIPPI1* gene by directly binding to the promoter of *AaIPPI1*. In transient AaZFP1 overexpressed *A. annua* plants, the artemisinin content was increased from 2.39 mg/g to 3.72 mg/g, which was 1.6 times the control [96]. AaSPL9 (SQUAMOSA promoter-binding protein like 9) can directly bind to the promoter of the *AaHD1* gene and activates the expression of the *AaHD1* gene. When AaSPL9 was overexpressed in *A. annua*, the density of glandular trichome increased by 45–60%, and the content of artemisinin went up by 33–60%—from 11.5 mg/g DW to 18.5 mg/g DW [97]. As a member of the MADS-box TF family, AaSEP4 is predominantly expressed in GSTs and activates the expression of TF AsGSW1 by directly binding to the CArG motif of the *AaGSW1* promoter. Overexpression of AaSEP4 remarkably up-regulated the transcription levels of the *AaGSW1*, *ADS*, *CYP71AV1*, *DBR2* and *ALDH1* genes, and compared with the wild type, the content of artemisinin increased by 19–72% in AaSEP4-overexpressed plants [98].

Table 2 and Table 3 summarize the current work of genetic engineering in *A. annua* to improve the generation of artemisinin. In general, the three strategies of “overexpression”, “suppression” and “global regulation” are adopted to achieve up-regulation of artemisinin accumulation. These strategies include overexpressing key enzymes that are upstream and downstream of the artemisinin biosynthesis pathway, blocking key genes in competitive metabolic pathways, and overexpressing or knocking-down TFs to globally regulate artemisinin biosynthesis. Based on the currently reported TFs of *A. annua*, the MYB TFs may act as key regulators, as they have the most binding sites in the promoters of artemisinin biosynthesis genes [116].

### 4.2. Genetic Engineering in Nicotiana Species

With *Nicotiana* as bioreactors, artemisinin had been successfully produced, but the yield of artemisinin was relatively low [21]. Tobacco had the advantages of large biomass and rapid growth, making it a suitable alternative plant for *A. annua* to study the heterologous biosynthesis of artemisinin [117] (Table 4).

When the *ADS* gene was expressed in *Nicotiana tabacum*, amorpha-4,11-diene, a precursor of artemisinin, can be produced in leaves at concentrations of 0.2–1.7 ng/g FW [118]. Through *Agrobacterium*-mediated transformation, five genes, including *FPPS*, *ADS*, *CYP71AV1*, *DBR2* and *ALDH1,* were transformed into *N. tabacum*, and about 4 μg/g FW of amorpha-4,11-diene was accumulated in transgenic tobacco leaves [119]. Five genes (*HMGR*, *ADS*/*mtADS*, *CYP71AV1*, *CPR* and *DBR2*) derived from the MVA pathway and the artemisinin biosynthesis pathway were constructed into the same vector and overexpressed in *N. tabacum*. The highest concentration of artemisinin was 6.8 μg/g DW in transgenic plants [120].

Van Herpen overexpressed the *HMGR*, *FPPS*, *ADS* and *CYP71AV1* genes of *A. annua* in *Nicotiana benthamiana* in order to produce the precursor of artemisinin—artemisinic acid in transgenic plants; however, artemisinic acid was not detected in the leaves, but the glycosylation product of artemisinic acid, artemisinic acid-12-β-diglucoside, was detected at a concentration of 39.5 µg/g FW [121]. This may be because when the key genes of artemisinin biosynthesis were heterologously expressed in *N. benthamiana*, glycosylated artemisinin precursors were mainly produced—with little free artemisinic acid or DHAA [122,123]. AaLTP3 (lipid transfer protein 3) and AaPDR2 (pleiotropic drug resistance 2) from *A. annua* prompted the accumulation of artemisinic acid and DHAA in leaf apoplasts of *N. benthamiana* and prevented the reflux of artemisinic acid and DHAA from the apoplast back into the cytoplasm [123].

**Table 4:** Comparison of artemisinin (or related products) biosynthesis in heterologous plants.

Gene Name	Plant Species	Expression System	Product Yield	Reference
*ADS*	*N. tabacum*	Nuclear	Amorpha-4,11-diene 1.7 ng/g FW	[118]
*FPPS*, *ADS*, *CYP71AV1*, *DBR2*, *ALDH1*	*N. tabacum*	Nuclear	Amorpha-4,11-diene 4 μg/g FW	[119]
*HMGR*, *ADS/mtADS*, *CYP71AV1*, *CPR*, *DBR2*	*N. tabacum*	Nuclear	Artemisinin 6.8 μg/g DW	[120]
*HMGR*, *FPPS*, *ADS*, *CYP71AV1*	*N. benthamiana*	Nuclear	Artemisinic acid-12-β-glucoside 39.5 μg/g FW	[121]
*AACT*^1^, *HMGS*^1^, *HMGR*^1^, *MVK*^1^, *PMK*^1^, *PMD*^1^, *IDI*^2^, *FPPS*^2^, *ADS*, *CYP71AV1*, *CPR*, *aad*A ^3^	*N. tabacum*	Chloroplast	Artemisinic acid 100 μg/g FW	[124]
*FPPS*, *ADS*, *CYP71AV1*, *CPR*, *CYPB5*, *ADH1*, *ALDH1*, *DBR2*	*N. tabacum*	Chloroplast and nuclear	Artemisinic acid 120 μg/g FW	[125]
*AACT*^1^, *HMGS*^1^, *HMGR*^1^, *MVK*^1^, *PMK*^1^, *PMD*^1^, *IDI*^2^, *FPPS*^2^, *ADS*, *CYP71AV1*, *CPR*, *DBR2*	*N. tabacum*	Chloroplast and nuclear	Artemisinin 0.8 mg/g DW	[126]
*ADS*, *CYP71AV1*, *ADH1*, *DBR2*, *ALDH1*	*P. patens*	Nuclear	Artemisinin 0.21 mg/g DW	[127]
*ADS*, *DBR2*, *ALDH1*	*P. patens*	Nuclear	Artemisinin 0.04 mg/g DW	[128]
*HMGR*, *ADS*, *CYP71AV1*, *CPR*, *DBR2*	*C. morifolium*	Nuclear	Artemisinin (Detect by GC-MS)	[129]

^1^ MVA pathway genes from yeast. ^2^ *IDI* and *FPPS* genes from *E. coli*. ^3^ *aad*A, an antibiotic selection marker gene.

To address the issue of glycosylation in tobacco, researchers attempted to target key genes of the artemisinin biosynthesis to different cellular compartments, such as chloroplasts, mitochondria and the nucleus. A new transformation method, COSTREL (combinatorial super transformation of transplastomic recipient lines), was adopted to transform the complete pathway of artemisinic acid biosynthesis into chloroplasts of *N. tabacum*, and the content of the artemisinic acid in transgenic plants reached 120 μg/g FW [125]. In 2014, Kumar’s laboratory transformed 12 genes of the MVA pathway and artemisinic acid biosynthesis pathway into chloroplasts of *N. tabacum* through a biolistic approach, but the yield of artemisinic acid was low (100 µg/g FW) in transgenic tobaccos and the growth of transgenic tobaccos was stunted. These results suggested that it is necessary to disperse the genes of the artemisinin biosynthetic pathway to different cellular compartments [124]. In 2016, Kumar’s laboratory separately transformed these two biosynthesis pathways into chloroplast and nucleus genomes of *N. tabacum*, respectively, which did not interfere with tobacco growth. First, six genes (*AACT*, *HMGS*, *HMGRt*, *MVK*, *PMK* and *PMD*) derived from the yeast MVA pathway were introduced into the chloroplast genome by a biolistic approach to increase the yield of IPP. Then, six key genes related to the artemisinin biosynthesis pathway (*IDI*, *FPPS*, *ADS*, *CYP71AV1*, *CPR* and *DBR2*) were transformed into the nuclear genome by the *Agrobacterium*-mediated method. Finally, DBR2, CPR and CYP71AV1 were transported into the chloroplast through the targeting of chloroplast transit peptides, and the artemisinin content in transgenic tobaccos reached 0.8 mg/g DW [126].

Although various methods have been used to improve the production of artemisinin in tobacco, due to the complexity of the heterologous expression and regulation of artemisinin biosynthesis genes, as well as the high level of glycosylation catalyzed by tobacco endogenous glycosyltransferase, the yield of artemisinin was still very low, but it is of great significance to study the heterologous biosynthesis of artemisinin in tobacco.

### 4.3. Genetic Engineering in Physcomitrella patens and Chrysanthemum morifolium

For more than two decades, *Physcomitrella patens* has become a model organism in plant biology, biotechnology and synthetic biology [130]. The *P. patens* genome was sequenced as early as 2008 [131], and the chromosome-level genomic data were released in 2018 [132]. In addition, *P. patens* had the advantages of a high homologous recombination rate, short growth cycle and large-scale culture, making it a green cell factory for metabolic engineering [133]. Different kinds of biopharmaceuticals, such as human complement factor H (FH), epidermal growth factor (EGF), hepatocyte growth factor (HGF), etc., have been successfully produced in *P. patens* [134]. The *TXS* (taxadiene synthase) gene from *T. brevifolia* was stably expressed in *P. patens*, and the content of taxadiene (the precursor of the anticancer drug paclitaxel) in the transgenic *P. patens* reached 0.05% FW [135].

In 2017, Ikram transformed five key genes of the artemisinin biosynthesis pathway (*ADS*, *CYP71AV1*, *ADH1*, *DBR2* and *ALDH1*) into *P. patens* through homologous recombination. A high initial yield of 0.21 mg/g DW artemisinin was detected in transgenic *P. patens* after only three days of culture [127]. In 2019, Ikram heterologously expressed these five artemisinin biosynthesis genes in *P. patens* with different combinations, and the results showed that both combinations of three different genes, ADS–CYP71AV1–ADH1 and ADS–DBR2–ALDH1, can produce artemisinin, indicating that there may be endogenous enzymes in *P. patens* that can complement the biosynthesis pathway of artemisinin. In ADS–DBR2–ALDH1 transgenic lines, 0.04 mg/g DW of artemisinin was accumulated, and artemisinin B was detected at 1.74 µg/g FW in the liquid medium [128] (Table 4).

As a moss without vascular tissue, *P. patens* has less glycosylase compared with higher plants. Using *P. patens* as a chassis to produce artemisinin, it was less likely to endogenously modify the intermediate metabolites of artemisinin; however, the yield of artemisinin in *P. patens* was very low, so new molecular tools needed to be developed for *P. patens* to improve artemisinin accumulation.

Both *Chrysanthemum morifolium* Ramat and *A. annua* belong to the *Compositae* family and are characterized by a high content of sesquiterpenes and their precursors [136]. Firsov et al. transformed five artemisinin pathway genes, including *HMGR*, *ADS*, *CYP71AV1*, *CPR* and *DBR2*, into *Chrysanthemum* by *Agrobacterium*-mediated transformation; artemisinin was detected in transgenic lines by both GC-MS and TLC [129,137]. The results suggested that artemisinin biosynthesis genes can be expressed in transgenic *Chrysanthemum* to generate artemisinin (Table 4).

## 5. Challenges and Perspectives

Malaria still poses a threat to human health, and artemisinin is the most effective drug to treat malaria. The precursor of artemisinin, artemisinic acid, was generated in yeast through metabolic engineering, which produced the semi-synthesis of artemisinin and reached the level of industrial application. This was a huge advance in the research process of artemisinin biosynthesis, thereby transferring artemisinin production from plant to yeast. With inexpensive carbon sources as substrates, artemisinic acid was synthesized by fermentation of engineering yeast, but subsequent extraction and chemical synthesis are required before artemisinic acid can be converted into artemisinin.

To date, the main source of artemisinin is extracted from *A. annua* planted in fields, but the small amount of artemisinin in *A. annua* can hardly meet the needs of the pharmaceutical market. The heterologous expression of artemisinin biosynthesis genes in *Nicotiana* and *Physcomitrella* can stably generate artemisinin, but the yield of artemisinin is very low. Therefore, we should focus on *A. annua* itself to improve the yield of artemisinin through new biotechnologies, such as gene editing or molecular design breeding, etc.

The biosynthesis of artemisinin occurs from plant to yeast and then back to plant—e.g., *A. annua*, *Nicotiana*, *P. patens*, etc. In terms of artemisinin biosynthesis, it remains to be studied whether it is more advantageous to produce artemisinin by fermentation in microorganisms, or to extract artemisinin from *A. annua* cultivated in fields. Artemisinin is usually generated in the leaves of *A. annua* and *Nicotiana* by field planting. On the contrary, yeast and *P. patens* are commonly used for fermentation, and algae can even be fermented by light. Through high-cost fermentation, the desired product can be harvested under controlled conditions with shorter production cycles. On the other hand, the cost of planting in fields is lower, but the production cycle is longer. The following are several questions and challenges worth considering:

1. The final step of artemisinin biosynthesis was generally considered to be a photo-oxidation reaction. Can the artemisinic acid produced by the fermentation of engineered yeast be used for photochemical reactions in a large-scale photoreactor to increase the production of artemisinin?

2. The biosynthesis of artemisinin was mainly carried out in the GSTs on the surface of leaves. In the future, artemisinin will be produced in all tissues of *A. annua* leaves by new gene editing biotechnology and will not be limited to GSTs.

3. The molecular modification and directed evolution of key enzymes in artemisinin biosynthesis are performed by protein engineering methods to improve the activities of these key enzymes and improve the content of artemisinin.

4. *Chlamydomonas reinhardtii* is a model organism for the study of photosynthesis, known as “green yeast”. The genetic background of *C. reinhardtii* is clear, and its genome was released in 2007. Moreover, the chloroplast and nuclear transformation methods of *C. reinhardtii* have been established. At present, there is no report on the biosynthesis of artemisinin in *C. reinhardtii*. Is it possible to transform the genes of artemisinin biosynthesis into *C. reinhardtii* through codon optimization and generate artemisinin by photo-fermentation?

5. Various TFs have been reported to regulate artemisinin biosynthesis. Are these TFs acting independently or cooperatively, and which TF is the most important?

## 6. Conclusions

Based on the analysis of the *A. annua* genome, the biosynthesis pathway of artemisinin and its regulatory mechanism were elucidated, and the genes of key enzymes as well as TFs involved in artemisinin biosynthesis were isolated and identified. With microorganisms or plants as the chassis, the biosynthesis pathway of artemisinin was reconstructed to increase the content of artemisinin or its precursors in order to obtain artemisinin in an efficient and low-cost manner. Such research strategies provided a paradigm for the use of synthetic biology methods to produce natural products of medicinal plants. Many natural products have important physiological activities and are important sources for the development of new drugs. We are confident that by reconstructing and modulating the biosynthesis pathway through the synthetic biology approach, the efficient biosynthesis of rare and important natural products can be achieved, thereby addressing both the quality and quantity issues. In summary, there are broad application prospects for the promotion of high-value natural products from laboratory to industrialization.

## Figures and Tables

**Table 1:** Metabolic engineering to enhance the production of amorpha-4,11-diene and artemisinic acid in *E. coli* or yeast.

Enzyme Name	Expression Mode	Gene Source	Host	Products	Reference
pMevT (atoB ^1^, HMGS, tHMGR) pMBIS (EGR12, EGR8, MVD1, idi ^2^, ispA ^3^)	Overexpression	Yeast	*E. coli*	Amorpha-4,11-diene 24 mg/L	[25]
pSOE4 (dxs, ippHP ^4^, ispA)	Overexpression	*E. coli*			
ADS	Overexpression	*A. annua*			
pAM45 (atoB1, HMGS, tHMGR, MK, PMK, PMD, idi ^2^, ispA ^3^,) pAM94 (ADS ^5^, MK)	Overexpression	Yeast	*E. coli*	Amorpha-4,11-diene 293 mg/L	[29]
pAM52 (atoB ^1^, mvaS, mvaA) ^6^ pMBIS (EGR12, EGR8, MVD1, idi ^2^, ispA ^3^)	Overexpression	Yeast	*E. coli*	Amorpha-4,11-diene 27.4 g/L	[27]
ADS	Overexpression	*A. annua*			
pAM92 (atoB ^1^, HMGS, tHMGR, EGR12, EGR8, EGR19, idi ^2^, ispA ^3^, ADS ^5^)	Overexpression	Yeast	*E. coli*	Artemisinic acid 105 mg/L	[28]
AMO, CPR	Overexpression	*A. annua*			
MVA pathway enzymes (EGR13, tHMGR, EGR12, EGR8, EGR20)	Overexpression	Yeast	Yeast	Artemisinic acid 100 mg/L	[18]
EGR9	Down-regulation	Yeast			
ADS, CYP71AV1, CPR	Overexpression	*A. annua*			
All enzymes of MVA pathway (EGR10, EGR13, tHMG1, EGR12, EGR8, EGR19, IDI1, EGR20)	Overexpression	Yeast	Yeast (CEN.PK2)	Amorpha-4,11-diene 40 g/L	[30]
EGR9	Down-regulation	Yeast			
ADS, CYP71AV1, CPR	Overexpression	*A. annua*			
Every gene of MVA pathway (EGR10, EGR13, tHMG1, EGR12, EGR8, EGR19, IDI1, EGR20)	Overexpression	Yeast	Yeast	Artemisinic acid 25 g/L	[31]
EGR9	Down-regulation	Yeast			
ADS, CYP71AV1, CPR1, CYB5, ADH1, ALDH1	Overexpression	*A. annua*			

^1^ atoB, acetoacetyl-CoA thiolase from *E. coli*. ^2^ idi, IPP isomerase from *E. coli*. ^3^ ispA, FPP synthase from *E. coli*. ^4^ ippHp, IPP isomerase from *Haematococcus pluvialis*. ^5^ ADS from *A. annua*. ^6^ pAM52 was the pMevT derivative, but HMGS and tHMGR were replaced by mvaS and mvaA from *S. aureus*.

## Data Availability

The data presented in this study are available on request from the corresponding author.

## References

[1] Yang L., Yang C., Li C., Zhao Q., Liu L., Fang X., Chen X.Y. (2016). Recent advances in biosynthesis of bioactive compounds in traditional Chinese medicinal plants. Sci. Bull..

[2] Zwenger S., Basu C. (2008). Plant terpenoids: Applications and future potentials. Microbiol. Mol. Biol. Rev..

[3] Nagegowda D.A., Gupta P. (2020). Advances in biosynthesis, regulation, and metabolic engineering of plant specialized terpenoids. Plant Sci..

[4] Alqahtani F.Y., Aleanizy F.S., El Tahir E., Alkahtani H.M., AlQuadeib B.T. (2019). Chapter Three—Paclitaxel. Profiles of Drug Substances, Excipients and Related Methodology.

[5] Hewlings S.J., Kalman D.S. (2017). Curcumin: A review of its effects on human health. Foods.

[6] Khan M., Ali S., Manghwar H., Saqib S., Ullah F., Ayaz A., Zaman W. (2022). Melatonin function and crosstalk with other phytohormones under normal and stressful conditions. Genes.

[7] Tu Y. (2011). The discovery of artemisinin (qinghaosu) and gifts from Chinese medicine. Nat. Med..

[8] World Health Organization World Malaria Report 2021. https://www.who.int/teams/global-malaria-programme/reports/world-malaria-report-2021.

[9] Wong Y.K., Xu C., Kalesh K.A., He Y., Lin Q., Wong W.S.F., Shen H.M., Wang J. (2017). Artemisinin as an anticancer drug: Recent advances in target profiling and mechanisms of action. Med. Res. Rev..

[10] Li J., Casteels T., Frogne T., Ingvorsen C., Honoré C., Courtney M., Huber K.V.M., Schmitner N., Kimmel R.A., Romanov R.A. (2017). Artemisinins target GABA_A_ receptor signaling and impair α cell identity. Cell.

[11] Zheng H., Colvin C.J., Johnson B.K., Kirchhoff P.D., Wilson M., Jorgensen-Muga K., Larsen S.D., Abramovitch R.B. (2017). Inhibitors of *Mycobacterium tuberculosis* DosRST signaling and persistence. Nat. Chem. Biol..

[12] Wallaart T.E., Pras N., Beekman A.C., Quax W. (2000). Seasonal variation of artemisinin and its biosynthetic precursors in plants of *Artemisia annua* of different geographical origin: Proof for the existence of chemotypes. Planta Med..

[13] Schmid G., Hofheinz W. (1983). Total synthesis of qinghaosu. J. Am. Chem. Soc..

[14] Zhu X., Liu X., Liu T., Wang Y., Ahmed N., Li Z., Jiang H. (2021). Synthetic biology of plant natural products: From pathway elucidation to engineered biosynthesis in plant cells. Plant Commun..

[15] Vranová E., Coman D., Gruissem W. (2013). Network analysis of the MVA and MEP pathways for isoprenoid synthesis. Annu. Rev. Plant Biol..

[16] Mercke P., Bengtsson M., Bouwmeester H.J., Posthumus M.A., Brodelius P.E. (2000). Molecular cloning, expression, and characterization of amorpha-4,11-diene synthase, a key enzyme of artemisinin biosynthesis in *Artemisia annua* L.. Arch. Biochem. Biophys..

[17] Teoh K.H., Polichuk D.R., Reed D.W., Nowak G., Covello P.S. (2006). *Artemisia annua* L. (Asteraceae) trichome-specific cDNAs reveal CYP71AV1, a cytochrome P450 with a key role in the biosynthesis of the antimalarial sesquiterpene lactone artemisinin. FEBS Lett..

[18] Ro D.K., Paradise E.M., Ouellet M., Fisher K.J., Newman K.L., Ndungu J.M., Ho K.A., Eachus R.A., Ham T.S., Kirby J. (2006). Production of the antimalarial drug precursor artemisinic acid in engineered yeast. Nature.

[19] Teoh K.H., Polichuk D.R., Reed D.W., Covello P.S. (2009). Molecular cloning of an aldehyde dehydrogenase implicated in artemisinin biosynthesis in *Artemisia annua*. Botany.

[20] Zhang Y., Teoh K.H., Reed D.W., Maes L., Goossens A., Olson D.J.H., Ross A.R.S., Covello P.S. (2008). The molecular cloning of artemisinic aldehyde Δ11(13) reductase and its role in glandular trichome-dependent biosynthesis of artemisinin in *Artemisia annua*. J. Biol. Chem..

[21] Ikram N.K.B.K., Simonsen H.T. (2017). A review of biotechnological artemisinin production in plants. Front. Plant Sci..

[22] Al-Khayri J.M., Sudheer W.N., Lakshmaiah V.V., Mukherjee E., Nizam A., Thiruvengadam M., Nagella P., Alessa F.M., Al-Mssallem M.Q., Rezk A.A. (2022). Biotechnological approaches for production of artemisinin, an anti-malarial drug from *Artemisia annua* L.. Molecules.

[23] Bryant L., Flatley B., Patole C., Brown G.D., Cramer R. (2015). Proteomic analysis of *Artemisia annua*—Towards elucidating the biosynthetic pathways of the antimalarial pro-drug artemisinin. BMC Plant Biol..

[24] Sun Y., Shang L., Zhu Q.H., Fan L., Guo L. (2022). Twenty years of plant genome sequencing: Achievements and challenges. Trends Plant Sci..

[25] Martin V.J.J., Pitera D.J., Withers S.T., Newman J.D., Keasling J.D. (2003). Engineering a mevalonate pathway in *Escherichia coli* for production of terpenoids. Nat. Biotechnol..

[26] Newman J.D., Marshall J., Chang M., Nowroozi F., Paradise E., Pitera D., Newman K.L., Keasling J.D. (2006). High-level production of amorpha-4,11-diene in a two-phase partitioning bioreactor of metabolically engineered *Escherichia coli*. Biotechnol. Bioeng..

[27] Tsuruta H., Paddon C.J., Eng D., Lenihan J.R., Horning T., Anthony L.C., Regentin R., Keasling J.D., Renninger N.S., Newman J.D. (2009). High-level production of amorpha-4,11-diene, a precursor of the antimalarial agent artemisinin, in *Escherichia coli*. PLoS ONE.

[28] Chang M.C.Y., Eachus R.A., Trieu W., Ro D.K., Keasling J.D. (2007). Engineering *Escherichia coli* for production of functionalized terpenoids using plant P450s. Nat. Chem. Biol..

[29] Anthony J.R., Anthony L.C., Nowroozi F., Kwon G., Newman J.D., Keasling J.D. (2009). Optimization of the mevalonate-based isoprenoid biosynthetic pathway in *Escherichia coli* for production of the anti-malarial drug precursor amorpha-4,11-diene. Metab. Eng..

[30] Westfall P.J., Pitera D.J., Lenihan J.R., Eng D., Woolard F.X., Regentin R., Horning T., Tsuruta H., Melis D.J., Owens A. (2012). Production of amorphadiene in yeast, and its conversion to dihydroartemisinic acid, precursor to the antimalarial agent artemisinin. Proc. Natl. Acad. Sci. USA.

[31] Paddon C.J., Westfall P.J., Pitera D.J., Benjamin K., Fisher K., McPhee D., Leavell M.D., Tai A., Main A., Eng D. (2013). High-level semi-synthetic production of the potent antimalarial artemisinin. Nature.

[32] Graham I.A., Besser K., Blumer S., Branigan C.A., Czechowski T., Elias L., Guterman I., Harvey D., Isaac P.G., Khan A.M. (2010). The genetic map of *Artemisia annua* L. identifies loci affecting yield of the antimalarial drug artemisinin. Science.

[33] Shen Q., Zhang L., Liao Z., Wang S., Yan T., Shi P., Liu M., Fu X., Pan Q., Wang Y. (2018). The genome of *Artemisia annua* provides insight into the evolution of asteraceae family and artemisinin biosynthesis. Mol. Plant.

[34] Ram M., Khan M.A., Jha P., Khan S., Kiran U., Ahmad M.M., Javed S., Abdin M.Z. (2010). HMG-CoA reductase limits artemisinin biosynthesis and accumulation in *Artemisia annua* L. plants. Acta Physiol. Plant..

[35] Nafis T., Akmal M., Ram M., Alam P., Ahlawat S., Mohd A., Abdin M.Z. (2011). Enhancement of artemisinin content by constitutive expression of the HMG-CoA reductase gene in high-yielding strain of *Artemisia annua* L.. Plant Biotechnol. Rep..

[36] Xiang L., Zeng L., Yuan Y., Chen M., Wang F., Liu X., Zeng L., Lan X., Liao Z. (2012). Enhancement of artemisinin biosynthesis by overexpressing *dxr*, *cyp71av1* and *cpr* in the plants of *Artemisia annua* L.. Plant Omics.

[37] Ma D., Li G., Zhu Y., Xie D. (2017). Overexpression and suppression of *Artemisia annua* 4-Hydroxy-3-methylbut-2-enyl diphosphate reductase 1 gene (*AaHDR1*) differentially regulate artemisinin and terpenoid biosynthesis. Front. Plant Sci..

[38] Ma D., Li G., Alejos-Gonzalez F., Zhu Y., Xue Z., Wang A., Zhang H., Li X., Ye H., Wang H. (2017). Overexpression of a type-I isopentenyl pyrophosphate isomerase of *Artemisia annua* in the cytosol leads to high arteannuin B production and artemisinin increase. Plant J..

[39] Matsushita Y., Kang W., Charlwood B.V. (1996). Cloning and analysis of a cDNA encoding farnesyl diphosphate synthase from *Artemisia annua*. Gene.

[40] Han J., Liu B., Ye H., Wang H., Li Z., Li G. (2006). Effects of overexpression of the endogenous farnesyl diphosphate synthase on the artemisinin content in *Artemisia annua* L.. J. Integr. Plant Biol..

[41] Banyai W., Kirdmanee C., Mii M., Supaibulwatana K. (2010). Overexpression of farnesyl pyrophosphate synthase (*FPS*) gene affected artemisinin content and growth of *Artemisia annua* L.. Plant Cell Tissue Organ Cult..

[42] Chen D., Ye H., Li G. (2000). Expression of a chimeric farnesyl diphosphate synthase gene in *Artemisia annua* L. transgenic plants via *Agrobacterium tumefaciens*-mediated transformation. Plant Sci..

[43] Wang H., Olofsson L., Lundgren A., Brodelius P. (2011). Trichome-specific expression of amorpha-4,11-diene synthase, a key enzyme of artemisinin biosynthesis in *Artemisia annua* L., as reported by a promoter-GUS fusion. Am. J. Plant Sci..

[44] Liao B., Shen X., Xiang L., Guo S., Chen S., Meng Y., Liang Y., Ding D., Bai J., Zhang D. (2022). Allele-aware chromosome-level genome assembly of *Artemisia annua* reveals the correlation between *ADS* expansion and artemisinin yield. Mol. Plant.

[45] Ma C., Wang H., Lu X., Wang H., Xu G., Liu B. (2009). Terpenoid metabolic profiling analysis of transgenic *Artemisia annua* L. by comprehensive two-dimensional gas chromatography time-of-flight mass spectrometry. Metabolomics.

[46] Yuan Y., Liu W., Zhang Q., Xiang L., Liu X., Chen M., Lin Z., Wang Q., Liao Z. (2015). Overexpression of artemisinic aldehyde Δ11 (13) reductase gene–enhanced artemisinin and its relative metabolite biosynthesis in transgenic *Artemisia annua* L.. Biotechnol. Appl. Biochem..

[47] Alam P., Abdin M.Z. (2011). Over-expression of HMG-CoA reductase and amorpha-4,11-diene synthase genes in *Artemisia annua* L. and its influence on artemisinin content. Plant Cell Rep..

[48] Han J., Wang H., Kanagarajan S., Hao M., Lundgren A., Brodelius P.E. (2016). Promoting artemisinin biosynthesis in *Artemisia annua* plants by substrate channeling. Mol. Plant.

[49] Shen Q., Chen Y., Wang T., Wu S., Xu L., Zhang L., Zhang F., Jiang W., Wang G., Tang K. (2012). Overexpression of the cytochrome P450 monooxygenase (*cyp71av1*) and cytochrome P450 reductase (*cpr*) genes increased artemisinin content in *Artemisia annua* (Asteraceae). Genet. Mol. Res..

[50] Chen Y., Shen Q., Wang Y., Wang T., Wu S., Zhang L., Lu X., Zhang F., Jiang W., Qiu B. (2013). The stacked over-expression of *FPS*, *CYP71AV1* and *CPR* genes leads to the increase of artemisinin level in *Artemisia annua* L.. Plant Biotechnol. Rep..

[51] Shi P., Fu X., Liu M., Shen Q., Jiang W., Li L., Sun X., Tang K. (2017). Promotion of artemisinin content in *Artemisia annua* by overexpression of multiple artemisinin biosynthetic pathway genes. Plant Cell Tissue Organ Cult..

[52] Aquil S., Husaini A.M., Abdin M.Z., Rather G.M. (2009). Overexpression of the HMG-CoA reductase gene leads to enhanced artemisinin biosynthesis in transgenic *Artemisia annua* plants. Planta Med..

[53] Tang K., Shen Q., Chen Y., Wang T., Wu S., Lu X. (2015). Overexpression DBR2 Gene Increased Artemisinin Content in *Artemisia annua* L.. China Patent Application.

[54] Tang K., Chen Y., Shen Q., Wang T., Wu S., Wang G. (2012). Overexpression ALDH1 Gene Increased Artemisinin Content in *Artemisia annua* L.. China Patent Application.

[55] Wang Y., Jing F., Yu S., Chen Y., Wang T., Liu P., Wang G., Sun X., Tang K. (2011). Co-overexpression of the *HMGR* and *FPS* genes enhances artemisinin content in *Artemisia annua* L.. J. Med. Plants Res..

[56] Lu X., Shen Q., Zhang L., Zhang F., Jiang W., Lv Z., Yan T., Fu X., Wang G., Tang K. (2013). Promotion of artemisinin biosynthesis in transgenic *Artemisia annua* by overexpressing *ADS*, *CYP71AV1* and *CPR* genes. Ind. Crops Prod..

[57] Zhang L., Jing F., Li F., Li M., Wang Y., Wang G., Sun X., Tang K. (2009). Development of transgenic *Artemisia annua* (Chinese wormwood) plants with an enhanced content of artemisinin, an effective anti-malarial drug, by hairpin-RNA-mediated gene silencing. Biotechnol. Appl. Biochem..

[58] Chen J., Fang H., Ji Y., Pu G., Guo Y., Huang L., Du Z., Liu B., Ye H., Li G. (2011). Artemisinin biosynthesis enhancement in transgenic *Artemisia annua* plants by downregulation of the β-caryophyllene synthase gene. Planta Med..

[59] Lv Z., Zhang F., Pan Q., Fu X., Jiang W., Shen Q., Yan T., Shi P., Lu X., Sun X. (2016). Branch pathway blocking in *Artemisia annua* is a useful method for obtaining high yield artemisinin. Plant Cell Physiol..

[60] Tang K., Shen Q., Yan T., Fu X. (2014). Transgenic approach to increase artemisinin content in *Artemisia annua* L.. Plant Cell Rep..

[61] Wani K.I., Choudhary S., Zehra A., Naeem M., Weathers P., Aftab T. (2021). Enhancing artemisinin content in and delivery from *Artemisia annua*: A review of alternative, classical, and transgenic approaches. Planta.

[62] Cai Y., Jia J.W., Crock J., Lin Z.X., Chen X.Y., Croteau R. (2002). A cDNA clone for β-caryophyllene synthase from *Artemisia annua*. Phytochemistry.

[63] Rydén A.M., Ruyter-Spira C., Quax W.J., Osada H., Muranaka T., Kayser O., Bouwmeester H. (2010). The molecular cloning of dihydroartemisinic aldehyde reductase and its implication in artemisinin biosynthesis in *Artemisia annua*. Planta Med..

[64] Ranjbar M., Naghavi M.R., Alizadeh H., Soltanloo H. (2015). Expression of artemisinin biosynthesis genes in eight *Artemisia* species at three developmental stages. Ind. Crops Prod..

[65] Rushton P.J., Somssich I.E., Ringler P., Shen Q.J. (2010). WRKY transcription factors. Trends Plant Sci..

[66] Chen X., Li C., Wang H., Guo Z. (2019). WRKY transcription factors: Evolution, binding, and action. Phytopathol. Res..

[67] Ma D., Pu G., Lei C., Ma L., Wang H., Guo Y., Chen J., Du Z., Wang H., Li G. (2009). Isolation and characterization of AaWRKY1, an *Artemisia annua* transcription factor that regulates the amorpha-4,11-diene synthase gene, a key gene of artemisinin biosynthesis. Plant Cell Physiol..

[68] Jiang W., Fu X., Pan Q., Tang Y., Shen Q., Lv Z., Yan T., Shi P., Li L., Zhang L. (2016). Overexpression of *AaWRKY1* leads to an enhanced content of artemisinin in *Artemisia annua*. BioMed Res. Int..

[69] Huang H., Xing S., Tang K., Jiang W. (2021). AaWRKY4 upregulates artemisinin content through boosting the expressions of key enzymes in artemisinin biosynthetic pathway. Plant Cell Tissue Organ Cult..

[70] Fu X., Peng B., Hassani D., Xie L., Liu H., Li Y., Chen T., Liu P., Tang Y., Li L. (2021). AaWRKY9 contributes to light- and jasmonate-mediated to regulate the biosynthesis of artemisinin in *Artemisia annua*. New Phytol..

[71] Chen T., Li Y., Xie L., Hao X., Liu H., Qin W., Wang C., Yan X., Wu-Zhang K., Yao X. (2021). AaWRKY17, a positive regulator of artemisinin biosynthesis, is involved in resistance to *Pseudomonas syringae* in *Artemisia annua*. Hortic. Res..

[72] Chen M., Yan T., Shen Q., Lu X., Pan Q., Huang Y., Tang Y., Fu X., Liu M., Jiang W. (2017). GLANDULAR TRICHOME-SPECIFIC WRKY 1 promotes artemisinin biosynthesis in *Artemisia annua*. New Phytol..

[73] Ma Y.N., Xu D.B., Yan X., Wu Z.K., Kayani S.I., Shen Q., Fu X.Q., Xie L.H., Hao X.L., Hassani D. (2021). Jasmonate- and abscisic acid-activated AaGSW1-AaTCP15/AaORA transcriptional cascade promotes artemisinin biosynthesis in *Artemisia annua*. Plant Biotechnol. J..

[74] Xie L., Yan T., Li L., Chen M., Ma Y., Hao X., Fu X., Shen Q., Huang Y., Qin W. (2021). The WRKY transcription factor AaGSW2 promotes glandular trichome initiation in *Artemisia annua*. J. Exp. Bot..

[75] Sun X., Wang Y., Sui N. (2018). Transcriptional regulation of bHLH during plant response to stress. Biochem. Biophys. Res. Commun..

[76] Ji Y., Xiao J., Shen Y., Ma D., Li Z., Pu G., Li X., Huang L., Liu B., Ye H. (2014). Cloning and characterization of AabHLH1, a bHLH transcription factor that positively regulates artemisinin biosynthesis in *Artemisia annua*. Plant Cell Physiol..

[77] Li L., Hao X., Liu H., Wang W., Fu X., Ma Y., Shen Q., Chen M., Tang K. (2019). Jasmonic acid-responsive AabHLH1 positively regulates artemisinin biosynthesis in *Artemisia annua*. Biotechnol. Appl. Biochem..

[78] Shen Q., Lu X., Yan T., Fu X., Lv Z., Zhang F., Pan Q., Wang G., Sun X., Tang K. (2016). The jasmonate-responsive AaMYC2 transcription factor positively regulates artemisinin biosynthesis in *Artemisia annua*. New Phytol..

[79] Xiang L., Jian D., Zhang F., Yang C., Bai G., Lan X., Chen M., Tang K., Liao Z. (2019). The cold-induced transcription factor bHLH112 promotes artemisinin biosynthesis indirectly via ERF1 in *Artemisia annua*. J. Exp. Bot..

[80] Yu Z.X., Li J.X., Yang C.Q., Hu W.L., Wang L.J., Chen X.Y. (2012). The jasmonate-responsive AP2/ERF transcription factors AaERF1 and AaERF2 positively regulate artemisinin biosynthesis in *Artemisia annua* L.. Mol. Plant.

[81] Shen Q., Huang H., Xie L., Hao X., Kayani S.-I., Liu H., Qin W., Chen T., Pan Q., Liu P. (2022). Basic helix-loop-helix transcription factors AabHLH2 and AabHLH3 function antagonistically with AaMYC2 and are negative regulators in artemisinin biosynthesis. Front. Plant Sci..

[82] Lu X., Zhang L., Zhang F., Jiang W., Shen Q., Zhang L., Lv Z., Wang G., Tang K. (2013). *AaORA*, a trichome-specific AP2/ERF transcription factor of *Artemisia annua*, is a positive regulator in the artemisinin biosynthetic pathway and in disease resistance to *Botrytis cinerea*. New Phytol..

[83] Tan H., Xiao L., Gao S., Li Q., Chen J., Xiao Y., Ji Q., Chen R., Chen W., Zhang L. (2015). *TRICHOME AND ARTEMISININ REGULATOR* 1 is required for trichome development and artemisinin biosynthesis in *Artemisia annua*. Mol. Plant.

[84] Zhang F., Fu X., Lv Z., Lu X., Shen Q., Zhang L., Zhu M., Wang G., Sun X., Liao Z. (2015). A basic leucine zipper transcription factor, AabZIP1, connects abscisic acid signaling with artemisinin biosynthesis in *Artemisia annua*. Mol. Plant.

[85] Shu G., Tang Y., Yuan M., Wei N., Zhang F., Yang C., Lan X., Chen M., Tang K., Xiang L. (2022). Molecular insights into AabZIP1-mediated regulation on artemisinin biosynthesis and drought tolerance in *Artemisia annua*. Acta Pharm. Sin. B.

[86] Shen Q., Huang H., Zhao Y., Xie L., He Q., Zhong Y., Wang Y., Wang Y., Tang K. (2019). The transcription factor AabZIP9 positively regulates the biosynthesis of artemisinin in *Artemisia annua*. Front. Plant Sci..

[87] Hao X., Zhong Y., Nützmann H.W., Fu X., Yan T., Shen Q., Chen M., Ma Y., Zhao J., Osbourn A. (2019). Light-induced artemisinin biosynthesis is regulated by the bZIP transcription factor AaHY5 in *Artemisia annua*. Plant Cell Physiol..

[88] Lv Z., Guo Z., Zhang L., Zhang F., Jiang W., Shen Q., Fu X., Yan T., Shi P., Hao X. (2019). Interaction of bZIP transcription factor TGA6 with salicylic acid signaling modulates artemisinin biosynthesis in *Artemisia annua*. J. Exp. Bot..

[89] Matías-Hernández L., Jiang W., Yang K., Tang K., Brodelius P.E., Pelaz S. (2017). AaMYB1 and its orthologue AtMYB61 affect terpene metabolism and trichome development in *Artemisia annua* and *Arabidopsis thaliana*. Plant J..

[90] Shi P., Fu X., Shen Q., Liu M., Pan Q., Tang Y., Jiang W., Lv Z., Yan T., Ma Y. (2018). The roles of *AaMIXTA1* in regulating the initiation of glandular trichomes and cuticle biosynthesis in *Artemisia annua*. New Phytol..

[91] Zhou Z., Tan H., Li Q., Li Q., Wang Y., Bu Q., Li Y., Wu Y., Chen W., Zhang L. (2020). *TRICHOME AND ARTEMISININ REGULATOR 2* positively regulates trichome development and artemisinin biosynthesis in *Artemisia annua*. New Phytol..

[92] Xie L., Yan T., Li L., Chen M., Hassani D., Li Y., Qin W., Liu H., Chen T., Fu X. (2021). An HD-ZIP-MYB complex regulates glandular secretory trichome initiation in *Artemisia annua*. New Phytol..

[93] Qin W., Xie L., Li Y., Liu H., Fu X., Chen T., Hassani D., Li L., Sun X., Tang K. (2021). An R2R3-MYB transcription factor positively regulates the glandular secretory trichome initiation in *Artemisia annua* L.. Front. Plant Sci..

[94] Wu Z., Li L., Liu H., Yan X., Ma Y., Li Y., Chen T., Wang C., Xie L., Hao X. (2021). AaMYB15, an R2R3-MYB TF in *Artemisia annua*, acts as a negative regulator of artemisinin biosynthesis. Plant Sci..

[95] Lv Z., Li J., Qiu S., Qi F., Su H., Bu Q., Jiang R., Tang K., Zhang L., Chen W. (2022). The transcription factors TLR1 and TLR2 negatively regulate trichome density and artemisinin levels in *Artemisia annua*. J. Integr. Plant Biol..

[96] Deng Y.A., Li L., Peng Q., Feng L.F., Yang J.F., Zhan R.T., Ma D.M. (2022). Isolation and characterization of AaZFP1, a C2H2 zinc finger protein that regulates the *AaIPPI1* gene involved in artemisinin biosynthesis in *Artemisia annua*. Planta.

[97] He Y., Fu X., Li L., Sun X., Tang K., Zhao J. (2022). AaSPL9 affects glandular trichomes initiation by positively regulating expression of *AaHD1* in *Artemisia annua* L.. Plant Sci..

[98] Chen T.T., Yao X.H., Liu H., Li Y.P., Qin W., Yan X., Wang X.Y., Peng B.W., Zhang Y.J., Shao J. (2022). MADS-box gene *AaSEP4* promotes artemisinin biosynthesis in *Artemisia annua*. Front. Plant Sci..

[99] Agarwal P.K., Agarwal P., Reddy M.K., Sopory S.K. (2006). Role of DREB transcription factors in abiotic and biotic stress tolerance in plants. Plant Cell Rep..

[100] Nakano T., Suzuki K., Fujimura T., Shinshi H. (2006). Genome-wide analysis of the ERF gene family in *Arabidopsis* and rice. Plant Physiol..

[101] Song X., Li Y., Hou X. (2013). Genome-wide analysis of the AP2/ERF transcription factor superfamily in Chinese cabbage (*Brassica rapa* ssp. pekinensis). BMC Genom..

[102] Lu X., Jiang W., Zhang L., Zhang F., Zhang F., Shen Q., Wang G., Tang K. (2013). AaERF1 positively regulates the resistance to *Botrytis cinerea* in *Artemisia annua*. PLoS ONE.

[103] Ma Y.N., Xu D.B., Li L., Zhang F., Fu X.Q., Shen Q., Lyu X.Y., Wu Z.K., Pan Q.F., Shi P. (2018). Jasmonate promotes artemisinin biosynthesis by activating the TCP14-ORA complex in *Artemisia annua*. Sci. Adv..

[104] Wang S., Zhang X., Li B., Zhao X., Shen Y., Yuan Z. (2022). Genome-wide identification and characterization of bZIP gene family and cloning of candidate genes for anthocyanin biosynthesis in pomegranate (*Punica granatum*). BMC Plant Biol..

[105] Yu Y., Qian Y., Jiang M., Xu J., Yang J., Zhang T., Gou L., Pi E. (2020). Regulation mechanisms of plant basic leucine zippers to various abiotic stresses. Front. Plant Sci..

[106] Jakoby M., Weisshaar B., Dröge-Laser W., Vicente-Carbajosa J., Tiedemann J., Kroj T., Parcy F. (2002). bZIP transcription factors in *Arabidopsis*. Trends Plant Sci..

[107] Choi H.I., Hong J.H., Ha J.O., Kang J.Y., Kim S.Y. (2000). ABFs, a family of ABA-responsive element binding factors. J. Biol. Chem..

[108] Foster R., Izawa T., Chua N.H. (1994). Plant bZIP proteins gather at ACGT elements. FASEB J..

[109] Jing F., Zhang L., Li M., Tang Y., Wang Y., Wang Y., Wang Q., Pan Q., Wang G., Tang K. (2009). Abscisic acid (ABA) treatment increases artemisinin content in *Artemisia annua* by enhancing the expression of genes in artemisinin biosynthetic pathway. Biologia.

[110] Pu G.B., Ma D.M., Chen J.L., Ma L.Q., Wang H., Li G.F., Ye H.C., Liu B.Y. (2009). Salicylic acid activates artemisinin biosynthesis in *Artemisia annua* L.. Plant Cell Rep..

[111] Dubos C., Stracke R., Grotewold E., Weisshaar B., Martin C., Lepiniec L. (2010). MYB transcription factors in *Arabidopsis*. Trends Plant Sci..

[112] Pesch M., Hülskamp M. (2009). One, two, three… models for trichome patterning in *Arabidopsis*?. Curr. Opin. Plant Biol..

[113] Yuan Y., Xu X., Luo Y., Gong Z., Hu X., Wu M., Liu Y., Yan F., Zhang X., Zhang W. (2021). R2R3 MYB-dependent auxin signalling regulates trichome formation, and increased trichome density confers spider mite tolerance on tomato. Plant Biotechnol. J..

[114] Dubos C., Le Gourrierec J., Baudry A., Huep G., Lanet E., Debeaujon I., Routaboul J.M., Alboresi A., Weisshaar B., Lepiniec L. (2008). MYBL2 is a new regulator of flavonoid biosynthesis in *Arabidopsis thaliana*. Plant J..

[115] Zhang Z., Zhang L., Liu Y., Shang X., Fang S. (2022). Identification and expression analysis of R2R3-MYB family genes associated with salt tolerance in *Cyclocarya paliurus*. Int. J. Mol. Sci..

[116] Hassani D., Fu X., Shen Q., Khalid M., Rose J.K.C., Tang K. (2020). Parallel transcriptional regulation of artemisinin and flavonoid biosynthesis. Trends Plant Sci..

[117] Molina-Hidalgo F.J., Vazquez-Vilar M., D’Andrea L., Demurtas O.C., Fraser P., Giuliano G., Bock R., Orzáez D., Goossens A. (2021). Engineering metabolism in *Nicotiana* species: A promising future. Trends Biotech..

[118] Wallaart T.E., Bouwmeester H.J., Hille J., Poppinga L., Maijers N.C.A. (2001). Amorpha-4,11-diene synthase: Cloning and functional expression of a key enzyme in the biosynthetic pathway of the novel antimalarial drug artemisinin. Planta.

[119] Zhang Y., Nowak G., Reed D.W., Covello P.S. (2011). The production of artemisinin precursors in tobacco. Plant Biotechnol. J..

[120] Farhi M., Marhevka E., Ben-Ari J., Algamas-Dimantov A., Liang Z., Zeevi V., Edelbaum O., Spitzer-Rimon B., Abeliovich H., Schwartz B. (2011). Generation of the potent anti-malarial drug artemisinin in tobacco. Nat. Biotechnol..

[121] Van Herpen T.W.J.M., Cankar K., Nogueira M., Bosch D., Bouwmeester H.J., Beekwilder J. (2010). *Nicotiana benthamiana* as a production platform for artemisinin precursors. PLoS ONE.

[122] Ting H.M., Wang B., Rydén A.M., Woittiez L., van Herpen T., Verstappen F.W.A., Ruyter-Spira C., Beekwilder J., Bouwmeester H.J., van der Krol A. (2013). The metabolite chemotype of *Nicotiana benthamiana* transiently expressing artemisinin biosynthetic pathway genes is a function of *CYP71AV1* type and relative gene dosage. New Phytol..

[123] Wang B., Kashkooli A.B., Sallets A., Ting H.M., de Ruijter N.C.A., Olofsson L., Brodelius P., Pottier M., Boutry M., Bouwmeester H. (2016). Transient production of artemisinin in *Nicotiana benthamiana* is boosted by a specific lipid transfer protein from *A. annua*. Metab. Eng..

[124] Saxena B., Subramaniyan M., Malhotra K., Bhavesh N.S., Potlakayala S.D., Kumar S. (2014). Metabolic engineering of chloroplasts for artemisinic acid biosynthesis and impact on plant growth. J. Biosci..

[125] Fuentes P., Zhou F., Erban A., Karcher D., Kopka J., Bock R. (2016). A new synthetic biology approach allows transfer of an entire metabolic pathway from a medicinal plant to a biomass crop. eLife.

[126] Malhotra K., Subramaniyan M., Rawat K., Kalamuddin M., Qureshi M.I., Malhotra P., Mohmmed A., Cornish K., Daniell H., Kumar S. (2016). Compartmentalized metabolic engineering for artemisinin biosynthesis and effective malaria treatment by oral delivery of plant cells. Mol. Plant.

[127] Khairul Ikram N.K.B., Beyraghdar Kashkooli A., Peramuna A.V., van der Krol A.R., Bouwmeester H., Simonsen H.T. (2017). Stable production of the antimalarial drug artemisinin in the moss *Physcomitrella patens*. Front. Bioeng. Biotech..

[128] Ikram N.K., Kashkooli A.B., Peramuna A., Krol A.R.v.d., Bouwmeester H., Simonsen H.T. (2019). Insights into heterologous biosynthesis of arteannuin B and artemisinin in *Physcomitrella patens*. Molecules.

[129] Firsov A., Pushin A., Motyleva S., Pigoleva S., Shaloiko L., Vainstein A., Dolgov S. (2021). Heterologous biosynthesis of artemisinin in *Chrysanthemum morifolium* Ramat. Separations.

[130] Reski R., Bae H., Simonsen H.T. (2018). *Physcomitrella patens*, a versatile synthetic biology chassis. Plant Cell Rep..

[131] Rensing S.A., Lang D., Zimmer A.D., Terry A., Salamov A., Shapiro H., Nishiyama T., Perroud P.-F., Lindquist E.A., Kamisugi Y. (2008). The *Physcomitrella* genome reveals evolutionary insights into the conquest of land by plants. Science.

[132] Lang D., Ullrich K.K., Murat F., Fuchs J., Jenkins J., Haas F.B., Piednoel M., Gundlach H., Van Bel M., Meyberg R. (2018). The *Physcomitrella patens* chromosome-scale assembly reveals moss genome structure and evolution. Plant J..

[133] King B.C., Vavitsas K., Ikram N.K.B.K., Schrøder J., Scharff L.B., Bassard J.-É., Hamberger B., Jensen P.E., Simonsen H.T. (2016). In vivo assembly of DNA-fragments in the moss, *Physcomitrella patens*. Sci. Rep..

[134] Reski R., Parsons J., Decker E.L. (2015). Moss-made pharmaceuticals: From bench to bedside. Plant Biotechnol. J..

[135] Anterola A., Shanle E., Perroud P.F., Quatrano R. (2009). Production of taxa-4(5),11(12)-diene by transgenic *Physcomitrella patens*. Transgenic Res..

[136] Yang L., Nuerbiye A., Cheng P., Wang J.H., Li H. (2017). Analysis of floral volatile components and antioxidant activity of different varieties of *Chrysanthemum morifolium*. Molecules.

[137] Firsov A., Mitiouchkina T., Shaloiko L., Pushin A., Vainstein A., Dolgov S. (2020). *Agrobacterium*-mediated transformation of *Chrysanthemum* with artemisinin biosynthesis pathway genes. Plants.

